# Design Strategies for Novel Lipid Nanoparticle for mRNA Vaccine and Therapeutics: Current Understandings and Future Perspectives

**DOI:** 10.1002/mco2.70414

**Published:** 2025-10-05

**Authors:** Xiaochi Li, Junli Li, Jiazheng Wei, Weixin Du, Cheng Su, Xiaobin Shen, Aihua Zhao, Miao Xu

**Affiliations:** ^1^ Division of Tuberculosis Vaccine and Allergen Products Institute of Biological Product Control, National Institutes For Food and Drug Control Beijing China; ^2^ State Key Laboratory of Drug Regulatory Science National Institutes for Food and Drug Control Beijing China; ^3^ Key Laboratory for Quality Research and Evaluation of Biological Products National Medical Products Administration (NMPA) Beijing China; ^4^ Key Laboratory of Research on Quality and Standardization of Biotech Products National Health Commission (NHC) Beijing China; ^5^ College of Life Sciences and Biopharmaceuticals Shenyang Pharmaceutical University Shenyang China

**Keywords:** adverse reactions, endosomal escape, lipid nanoparticle, manufacture and quality control, mRNA vaccine, targeted, therapeutics

## Abstract

Messenger RNA (mRNA) vaccines have revolutionized infectious disease prevention and cancer immunotherapy due to their rapid development, potent immunogenicity, and flexible design. Central to the clinical success of mRNA vaccines, lipid nanoparticles (LNPs) function as efficient, nonviral delivery systems capable of protecting mRNA and facilitating its uptake by target cells. Recent advances have demonstrated that LNP‐formulated mRNA vaccines and therapeutics elicit robust immune responses and confer effective protection against a broad spectrum of pathogens, including viruses and bacteria. Moreover, LNP‐based therapies have shown promising therapeutic efficacy in various cancers and rare diseases, as evidenced by both preclinical models and clinical trials. This review provides a comprehensive overview of the key components, structural features, and preparation technologies of LNPs. It further discusses ongoing challenges in LNP design, such as delivery efficiency, tissue targeting, and safety, and proposes rational strategies to address these limitations. Additionally, recent progress in the analytical methods used to characterize the critical quality attributes of LNPs is highlighted. This review aims to guide the rational design of next‐generation LNPs and to support the broader application of mRNA‐based vaccines and therapeutics.

## Graphical abstract

This review summarizes strategies to optimize lipid nanoparticles (LNPs) for improved targeting, endosomal escape, and safety. It highlights mRNA design, alternative delivery systems, and recent therapeutic advances across infectious diseases, cancer, and drug applications. Fundamental components, synthetic methods, and analytical tools are discussed, providing guidance for next‐generation LNP design and their transition toward precision‐based therapies.

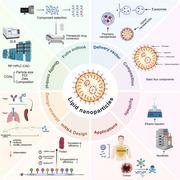

## 1 Introduction

Over the past few decades, the development of nucleic acid therapeutics has been hampered by the lack of efficient delivery systems. The advent of lipid nanoparticles (NPs) (LNPs) technology has addressed this major bottleneck, enabling the clinical translation of messenger RNA (mRNA)‐based therapies. Building on early foundational studies that demonstrated mRNA's capacity to direct protein synthesis in vivo, LNP‐enabled delivery has emerged as a cornerstone for the rapid and scalable deployment of mRNA vaccines [1, 2]. The COVID‐19 pandemic catalyzed unprecedented progress in mRNA vaccine development. Pfizer/BioNTech's BNT162b2 and Moderna's mRNA‐1273 received emergency use authorization within 1 year of SARS‐CoV‐2 genome publication, demonstrating efficacy rates of 95 and 94.1%, respectively. These milestones established LNP‐formulated mRNA vaccines as a revolutionary tool against infectious diseases [3, 4]. Importantly, the scope of LNP applications now extends well beyond infectious disease prevention. Recent advances highlight their potential in cancer immunotherapy, exemplified by Moderna's KRAS mRNA vaccine (NCT03948763) currently under clinical evaluation for solid tumors, as well as other tumor‐associated antigen (TAA)‐targeted mRNA vaccines entering early‐phase oncology trials [5]. In addition, LNP platforms are being developed for the treatment of rare genetic disorders, including cystic fibrosis and metabolic diseases, by delivering functional proteins that are otherwise absent or defective. This shift underscores a transition from prophylactic vaccines to programmable and precision therapeutics, positioning LNP technologies as versatile modalities in modern medicine.

mRNA technologies, featuring advanced lipid components and strategies for controlling immunogenicity, such as nucleoside base modification, are expanding into broader therapeutic areas, including cancer immunotherapy, rare diseases, and autoimmune disorders. Although target antigenic proteins can be expressed through in vivo injection of mRNA molecules, the characteristics of mRNA—such as their susceptibility to enzymatic degradation, instability, and short half‐life—make it challenging to achieve pharmacological effects [6, 7, 8]. The stability and translational efficiency of mRNA can be improved through various strategies, including base chemical modifications (e.g., substituting uracil nucleoside with N1‐methylpseudouridine nucleoside), optimizing species‐specific codons, and enhancing the 5′ cap structure and 3′ poly(A) tail element [9, 10, 11, 12, 13, 14]. However, the clinical application of mRNA encounters three delivery barriers: (1) mRNA's strong negative charge hinders its penetration of the cell membrane for translation; (2) RNA enzymes in the body rapidly degrade nucleic acids; and (3) lysosomes can sequester the mRNA, preventing timely release into the cytoplasm to bind ribosomes [15]. Overcoming these biological barriers requires the development of efficient delivery systems that address the challenges of charge shielding, nucleic acid protection, and intracellular release, which are essential for the successful translational application of mRNA vaccines and therapeutic [16].

Delivery vectors commonly used in vaccine development are categorized as viral (adenoviruses, adeno‐associated viruses, retroviruses, and lentiviruses) and nonviral (LNPs, polymers, and exosomes) [6, 17]. Viral vectors are limited by the risk of genomic integration and complex production processes [18]. Polymer and exosome vectors are hindered by their restricted drug delivery capacity and challenges in achieving large‐scale production. LNPs are widely utilized not only in infectious disease prevention but also in therapeutic applications, due to their good biocompatibility, high delivery efficiency, and safety.

This article reviews other delivery systems, basic components of LNPs, including their preparation methods, the application of mRNA–LNP vaccines and therapeutics, molecular design strategies of mRNA, structural optimization of lipids, and quality control measures in the LNPs production process. The primary objective is to provide theoretical support for designing LNPs with enhanced delivery performance, thereby expanding the application potential of LNP‐based mRNA vaccines and therapeutics.

## 2 Delivery Systems

Although LNPs have emerged as the leading platform for delivery due to their high encapsulation efficiency, biocompatibility, and scalable manufacturing, they are not the only nanocarrier system under active investigation. Alternative delivery vehicles, such as exosomes, polymeric NPs (PNPs), hybrid NP systems, and liposomes, offer unique benefits including structural versatility, natural biomimicry, and multifunctionality. However, these alternatives often face challenges related to production complexity, stability, safety, and efficient cytosolic delivery. In contrast, LNPs combine well‐established manufacturing processes with robust physicochemical properties and proven clinical success, positioning them as the current gold standard in mRNA prevention and therapeutics.

### 2.1 Exosomes

Exosomes have a negatively charged surface and are primarily composed of phosphatidylcholine (10–30%), cholesterol (0–42%), phosphatidylserine (PS) (6–15%), and sphingomyelin (8–23%). They also contain smaller proportions of phosphatidylethanolamine, phosphatidylinositol, and ceramides [19, 20]. Derived from biological fluids like cell culture supernatants, blood, urine, and breast milk, the clinical translation of exosome‐based nucleic acid delivery systems faces several challenges, including large‐scale production issues associated with cell culture methods and purification strategies [21]. Moreover, the lack of standardized critical quality attributes (CQAs) during exosome manufacturing further complicates regulatory approval and quality assessment. Insufficient knowledge of exosome formulations and optimal storage conditions has resulted in low encapsulation efficiency of nucleic acids, introducing further uncertainty about their clinical applicability [22, 23]. Meanwhile, LNPs, with established manufacturing protocols, high encapsulation efficiency, and tunable targeting capabilities, have achieved significant clinical success, highlighting their advantages over exosome‐based delivery systems.

### 2.2 Polymeric NPs

PNPs are nanoscale delivery systems constructed from natural or synthetic polymers, including poly (lactic‐co‐glycolic acid) (PLGA), polycaprolactone, polyethylene glycol (PEG), polyethylenimine (PEI), and chitosan [24]. PLGA‐based NPs have been extensively employed to modulate the release kinetics of small‐molecule drugs, prolonging therapeutic duration [25]. Cationic polymers, such as PEI and polyamidoamine, complex with small interfering RNA (siRNA) or mRNA through electrostatic interactions to enhance cellular uptake and endocytosis [26]. Chitosan‐based NPs have been investigated for oral vaccine delivery to enhance mucosal immune responses [27].

Despite these advantages, PNPs face several critical limitations in nucleic acid delivery. High‐charge‐density polymers like PEI cause substantial cytotoxicity and hemolytic risk. Moreover, polymer–nucleic acid complexes often lack stability, particularly for delivering long‐stranded mRNA molecules. PNPs also lack effective endosomal escape mechanisms, such as membrane fusion, crucial for cytosolic release. Moreover, complex polymer synthesis and considerable batch‐to‐batch variability pose significant obstacles to clinical translation and large‐scale manufacturing [28, 29]. Conversely, LNPs, composed of naturally derived or cell membrane–mimicking lipids, offer superior biocompatibility, high encapsulation efficiency, and robust physicochemical stability. LNPs also benefit from well‐established microfluidic manufacturing platforms, enabling scalable and reproducible production suitable for clinical and industrial use.

### 2.3 Hybrid NPs

Hybrid NP systems combine two or more materials, such as lipids with polymers, inorganic NPs, or proteins. For example, lipid–polymer hybrid NPs comprise a polymeric core—such as PLGA—that provides structural stability, encapsulated within a lipid shell to improve biocompatibility and mRNA encapsulation. Lipid‐coated gold or silica NPs enhance imaging, thermal responsiveness, and reactive oxygen species (ROS) modulation [30]. Nevertheless, these hybrid NP systems face challenges like interfacial stability, standardization, scalability, and long‐term biosafety. Similar to exosomes and polymeric NPs, hybrids require complex synthesis protocols and face challenges in achieving batch‐to‐batch consistency, complicating quality control and clinical translation. LNPs have become widely adopted in tumor and gene therapy, and infectious disease prevention due to their biocompatibility, delivery efficiency, and safety profile [31].

### 2.4 Liposomes

Liposomes are spherical vesicles composed of one or more phospholipid bilayers enclosing an aqueous core and have long been utilized as nanocarriers for small molecules, peptides, proteins, and nucleic acids [32]. Their biocompatibility, structural versatility, and ability to encapsulate both hydrophilic and hydrophobic drugs have made liposomes one of the most clinically validated drug delivery systems, exemplified by doxorubicin‐loaded liposomes [33]. For nucleic acid delivery, cationic or ionizable lipid‐containing liposomes can electrostatically bind negatively charged RNA molecules, facilitating cellular uptake and protection against enzymatic degradation.

However, conventional liposomes face several limitations for mRNA delivery, including relatively low encapsulation efficiency, instability during storage, and insufficient endosomal escape capacity compared with advanced LNPs. Additionally, large‐scale reproducibility remains challenging [34]. Despite these drawbacks, liposomes remain an important platform for therapeutic delivery, and continued advances in liposome engineering may bridge the gap toward more efficient nucleic acid formulations.

In summary, while exosomes, PNPs, hybrid NPs, and liposomes each present unique features that may complement or enhance mRNA delivery, their clinical translation remains limited by manufacturing complexity, insufficient endosomal escape efficiency, and concerns regarding safety and reproducibility. Compared with these alternatives, LNPs maintain a clear advantage with their clinically validated performance, efficient intracellular delivery mechanisms, and scalable production platforms. Subsequently, the basic composition and preparation process of LNPs are elaborated in detail.

## 3 LNP Composition

LNPs were initially employed as carriers to deliver siRNA. In 2002, when Tuschl et al. [35] synthesized siRNA in vitro, it entered the cytoplasm and attached to the Argonaute protein and Dicer enzyme to create an RNA‐induced silencing complex. In 2006, Zimmermann et al. [36] intravenously injected crab‐eating rhesus monkeys with LNP‐encapsulated siRNAs, achieving sustainable, dose‐dependent silencing of mRNA expression and demonstrating the feasibility of LNP delivery of siRNAs in nonhuman primates. The success of siRNA delivery demonstrated the potential of LNPs as nonviral vectors for selective gene expression regulation in higher animal models. In 2018, the United States Food and Drug Administration (US FDA) approved Onpattro (patisiran, ALN‐TTR02, Alnylam), a siRNA drug for hereditary transthyretin amyloidosis, leveraging breakthroughs in LNP technology to become the first four‐component LNP‐based siRNA product on the market. As LNP delivery technology matures, its application in mRNA vaccines is becoming increasingly widespread. With the global spread of COVID‐19, mRNA vaccines are being integrated with this technology [37, 38]. Many research teams are studying the structure and function of LNP components to develop products with improved delivery performance for clinical research.

**FIGURE 1:**
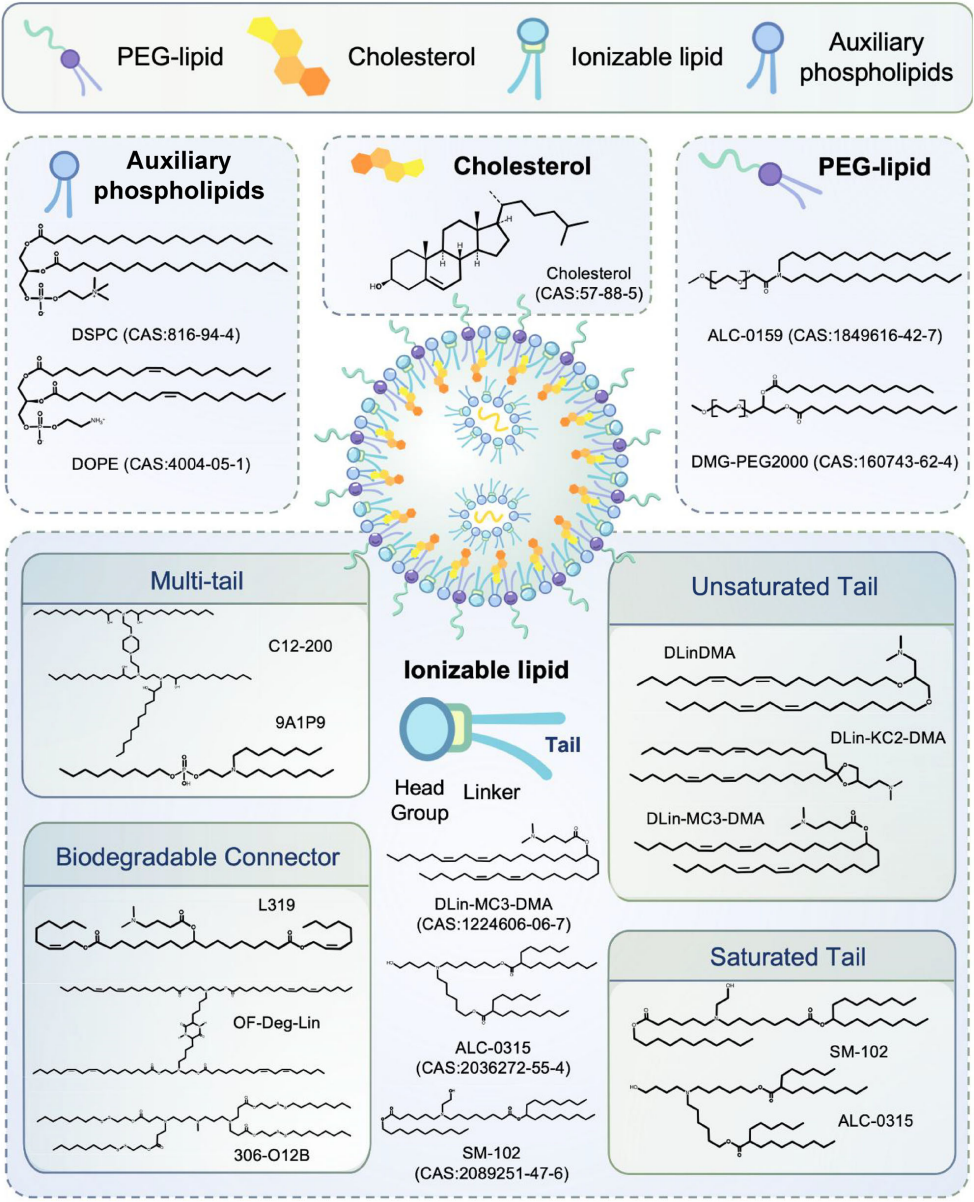
Schematic diagram of the four‐component structure of LNPs. Ionizable lipids, auxiliary phospholipids, PEGylated lipids, and cholesterol constitute the basic structure of LNPs; patisiran, BNT162b2, and mRNA‐1273 in the marketed products use DLin‐MC3‐DMA, ALC‐0315, and SM‐102 ionizable lipids, respectively. LNPs in the marketed preventive mRNA vaccines select DSPC auxiliary lipids. Schematic representation of cholesterol structure. BNT162b2 and mRNA‐1273 use ALC‐0159 and PEG‐2000‐DMG as PEGylated lipids, respectively; CAS: Chemical Substance Authority identification number. Schematic structure of ionizable lipids. mRNA ionizable lipids are classified into four types according to the linkage bonds to the tail moiety and structure: unsaturated tail‐chain lipids (DLinDMA, DLin‐KC2‐DMA, DLin‐MC3‐DMA), saturated‐tail‐chain lipids (SM‐102, ALC‐0315), multitailed (number of branched chains in the tail > 2) lipids (C12‐200, 9A1P9), and degradable lipids (L319, OF‐Deg‐Lin, 306‐O12B) (created in https://BioRender.com).

The components and structure of LNPs (Figure 1) include:

1. Ionizable lipids bind to negatively charged mRNA molecules through electrostatic interaction, facilitating endocytosis and delivery efficiency.
2. Auxiliary phospholipids stabilize the LNP bilayer, enhancing stability and storage time.
3. Cholesterol on the LNP surface regulates lipid membrane fluidity, improving stability.
4. PEG lipids on the LNP surface shield residual charge and prolong circulation time in the organism.

### 3.1 Ionizable Lipids: Balancing Charge Dynamics and Delivery Properties

Buschmann et al. [39] reported that the negative charge of LNPs is not conducive to their accumulation in muscles and lymph nodes, which is necessary to trigger an immune response. The negatively charged LNP tends to be widely distributed in vivo, increasing the risk of adverse effects such as chills and fever in mice. The efficacy of the vaccine is closely related to the charge carried by LNPs. Although cationic lipids are frequently employed to deliver nucleic acid substances as key components of LNPs, their toxicity and short half‐life can adversely affect drug efficacy in vivo [40]. Currently, pH‐sensitive lipids are preferred as they remain neutral under physiological conditions after being assembled into LNPs, but they become positively charged in the acidic endosomes of the nucleus [41]. They offer low toxicity, facilitate endosomal escape, and thus enhance the in vivo efficacy of the loaded drug [42, 43, 44]. Typically, this class of lipids constitutes 30–50% of the total LNP composition, with their structure directly influencing their function [45].

Ionizable lipids generally have a hydrophilic head group, a linkage bond, and a hydrophobic tail group comprising amine groups (quaternary and tertiary amines), heterocyclic rings, and guanidine groups (Figure 1) [46, 47, 48]. The head group, typically positively charged, binds mRNA via electrostatic interactions, protecting it from nucleases. Additionally, the head group fuses with the target cell membrane, aiding cell entry [47]. The linker bonds act as “bridges” between the head and tail of the ionizable lipids and are categorized as nondegradable (ethers and carbamates) or degradable (esters, amides, and thiols). Nondegradable linkages have a high transfection efficiency but increase lipid cytotoxicity due to overstabilization. Meanwhile, degradable linkers are preferred when designing novel lipid structures for their rapid clearance and reduced side effects following multiple administrations [49, 50]. Hydrophobic tails comprise saturated or unsaturated hydrocarbon chains, branched structures, and steroid derivatives, enhancing LNP stability by supporting the phospholipid bilayer structure.

Unsaturated hydrocarbon chains and branched structures enhance membrane fluidity and transfection efficiency. Cholesterol derivatives improve membrane rigidity and structural integrity when used as ionizable lipids tails. Heyes et al. [51] synthesized four ionizable lipids in vitro with trailing alkyl branched chains containing zero (bis (2‐methacryloyl)‐oxyethyl‐disulfide), one (1,2‐dioleyloxy‐3‐dimethylamino‐propane), two (1,2‐diloinoleyloxy‐3‐dimethylaminopropane [DLinDMA]), and three (2‐dilinolenyloxy‐N,N‐dimethyl‐3‐aminopropane) unsaturated double bonds. The findings indicated that the saturation of the ionizable lipid tails influenced the delivery efficiency of LNP, with saturated tails correlating positively with the cellular uptake capacity. Higher saturation of ionizable lipid tails improves cellular uptake but reduces endosomal escape capacity, affecting delivery efficiency.

Hope et al. [52] uniformly adopted a structure analogous to the DLinDMA tail structure within the same alkyl chain class, opting for a ketocyclic structure as the connecting bond. They constructed an ionizable lipid library by designing head group structures composed of varying numbers of methylene groups. An ionizable lipid with two methylene groups in the head (DLin‐KC2‐DMA) exhibited the highest delivery efficiency, and DLin‐KC2‐DMA lipids demonstrated favorable tolerance and delivery performance in rodents and nonhuman primates. The research team further optimized the head group of ionizable lipids based on the structure of DLin‐KC2‐DMA and synthesized 53 novel ionizable lipids. DLin‐MC3‐DMA, with three methylene groups in the head group, a degradable ester linkage, and unsaturated tails, exhibited the highest in vivo delivery efficacy, with an optimal p*K*_a_ range of 6.2–6.5 [53].

Alnylam conducted a Phase III clinical trial (NCT01960348) in the United States, involving 225 adults aged 18–85 years, which demonstrated that patisiran effectively treats hereditary thyrotropic transprotein amyloidosis [54]. The successful market launch of patisiran was significantly attributed to the ionizable lipid DLin‐MC3‐DMA composition based on LNPs. Pardi et al. [55] reported that SM‐102 (mRNA‐1273) and ALC‐0315 (BNT162b2) share structural similarities, containing saturated alkyl chains in the tails, degradable ester bonds as connecting elements, and hydroxyl groups at the ends of their head groups [56]. These characteristics reduce head group hydration and enhance hydrogen bonding to nucleic acids, thereby improving the transfection capacity [57, 58]. Despite these similarities, they differ in the number of sub‐methyl groups in the tail alkyl branched chain and head group. Consequently, SM‐102 demonstrates superior potency and stability, inducing higher antibody titers in BALB/C mice. The successful conversion of these two ionizable lipids into products further affirmed the feasibility of employing saturated alkyl chains.

Song et al. [59] designed an LNP featuring a novel ionizable lipid (4N4T) with four tertiary amine groups and four hydrophobic chains. This LNP encapsulated the 4N4T‐DS mRNA vaccine encoding the full‐length S protein sequence of SARS‐CoV‐2. Experimental results indicated that the mRNA translation efficiency of 4N4T–LNP was markedly higher than that of SM‐102–LNP, with the 4N4T‐DS mRNA vaccine inducing higher neutralizing antibody titers. The type of neutralizing antibody produced was related to the chemical structure of the ionizable lipid. In conclusion, the design of the head group structure and the modification of the added groups of ionizable lipids can promote the delivery of LNP, the selection of degradable linkages can reduce the toxicity of lipids, and the different types of tail structures can also affect the interaction between LNP and the cell membrane. Consequently, when formulating new ionizable lipids, it is anticipated that integrating components with diverse structural functions will enhance the delivery efficacy of LNP.

### 3.2 Auxiliary Phospholipids: Regulating Membrane Stability and Biodistribution

Auxiliary lipids constitute 10–20% of LNPs, with 1,2‐dioleoyl‐sn‐glycero‐3‐phosphoethanolamine (DOPE) a prevalent example. Other examples include 1,2‐dioleoyl‐sn‐glycero‐3‐phosphocholine (DOPC) and 1,2‐distearoyl‐sn‐glycero‐3‐phosphocholine (DSPC). DOPE contains ethanolamine phosphate and two unsaturated alkyl chains connected by ester bonds. This structural characteristic stabilizes the hexagonal II (H_II_) phase, crucial for membrane fusion, transmembrane transport, and lipid flip‐flop movement [60, 61].

In 2020, Ermilova and Swenson [62] utilized DLin‐MC3‐DMA as an ionizable lipid model in atomic molecular dynamics simulations, revealing that under equivalent DOPE and DOPC concentrations, DLin‐MC3‐DMA predominantly localizes within the DOPC lipid bilayer, enhancing membrane stability. Conversely, Lin‐MC3‐DMA exhibits a propensity to accumulate on the DOPE surface. This distribution disparity can be attributed to proton transfer disruption around the head groups, which impacts the LNPs’ transfection efficiency. DSPC differs structurally from DOPE and DOPC, containing saturated alkyl chains and possessing a high melting temperature suitable for constructing highly stable LNPs; however, an overly stable LNP structure is not conducive to endosomal escape [63, 64, 65]. This highlights the tradeoff between cellular‐level stability and transfection potency during transformation studies.

Auxiliary lipids also influence the biodistribution of LNP‐delivered drugs. For instance, LNPs composed of DOPE tend to accumulate in the liver, whereas those composed of DSPC tend to accumulate in the spleen [61]. Importantly, auxiliary lipids are generally nontoxic, nonimmunostimulatory, and nonhemolytic, ensuring minimal interference with LNP delivery. Adjuvant lipids regulate LNP membrane stability and fluidity and do not carry an intrinsic positive charge, thus reducing immune responses to LNP [66]. Notably, Onpattro, Spikevax, and Comirnarty employ DSPC as an adjuvant lipid. Consequently, the rationale design of adjuvant lipids will continue to be a focal point in future studies.

### 3.3 Cholesterol: Membrane Fluidity Regulation and Delivery Potentiation

Natural sources of cholesterol are abundant, and cholesterol is a key component of animal cell membranes, typically comprising 20–50% of LNPs [61, 67, 68]. The positioning of cholesterol on the LNP surface influences membrane fluidity, stability, delivery efficacy, and circulating half‐life [69]. In 2019, Paunovska et al. [70] successfully created an LNP database comprising nine cholesterol analogs. They reported that oxidatively modified cholesterol efficiently delivers mRNAs to cells within the liver microenvironment [70]. Furthermore, the hydrocarbon tail of cholesterol exhibits enhanced tolerance to oxidative modification compared with the cholesterol ring.

In 2024, Jung et al. [71] developed an ionizable cholesterol analog, 3β[L‐histidinamide‐carbamoyl] cholesterol (Hchol), as a substitute for cholesterol in LNP assembly. Results from in vivo efficacy experiments revealed that the imidazole moiety of Hchol promotes endosomal escape and increases the delivery potency and stability of LNPs. Xie et al. and Li et al. replaced the cholesterol component with methylprednisolone (MP) to prepare novel MP–LNPs. MP retained its anti‐inflammatory properties while avoiding systemic exposure to the drug to mitigate side effects. Furthermore, delivering MP–LNP‐encapsulated mRNA encoding C3 transferase (MP–LNP–C3) to injury sites effectively repairs damaged neural networks [72]. The precise mechanism by which cholesterol, which is inert to lipid responses, affects LNP recognition by target cells is unclear. A comprehensive analysis of impurities in raw cholesterol materials is crucial for LNP quality control.

### 3.4 PEG Lipids: Balancing Stability and Immunogenicity

The PEG lipids in LNPs typically constitute approximately 1.5%. They are predominantly distributed on the surface of LNPs, impeding LNP aggregation and enhancing their stability. PEGylated lipids also create a spatial barrier on the surface of LNPs, hindering their clearance by macrophages and prolonging their circulation time and half‐life. However, PEGylated lipids may trigger the production of antibodies that can lead to pseudo‐hypersensitivity activated by complement, contributing to adverse reactions caused by mRNA vaccines [73, 74].

In 2010, Akinc et al. [75] demonstrated that apolipoprotein E (ApoE) binds LNPs and mediates the delivery of LNPs to hepatocytes via low‐density lipoprotein receptors (LDLRs). Subsequent studies found that hydrophobic interactions between PEGylated lipids and biofilms correlate with alkyl chain length [75, 76, 77]. Meanwhile, in 2020, Suzuki et al. [77] reported that the shedding rate of PEGylated lipids in LNPs inversely relates to the length of the alkyl chain, with faster‐shedding lipid NPs inducing IgM antibody titers. Hence, rationally designing PEGylated lipids are imperative to mitigate adverse effects.

Furthermore, the PEGylated lipid content influences delivery efficacy, with comparatively low levels of PEGylated lipids exhibiting higher delivery potency [78]. PEG‐sized lipids effectively enhance the delivery potency of LNPs after nebulization [79]. Additionally, they can be biocoupled with ligands or macromolecules to serve specific functions. For example, Parhiz et al. [80] utilized the N‐Succinimidyl‐S‐acetylthioacetate‐maleimide (SATA‐maleimide) coupling technique to couple antibodies to PEGylated lipids, resulting in efficient drug delivery to the lungs. Meanwhile, Singh et al. [81] demonstrated the potential of PEGylated lipids for delivering LNPs via aerosolization by coupling hyaluronic acid to PEGylated lipids, achieving targeted drug delivery to ovarian cancer cells. The functionalization of PEGylated lipids is essential for improving LNP delivery efficacy, targeting precision, and stability. However, further investigation is necessary to understand the allergic reactions associated with PEGylated lipids. Thus, it is essential to explore the mechanisms underlying the action of PEGylated lipids and their role in triggering allergic reactions.

Once the key structural components of LNPs are defined, selecting an appropriate manufacturing process becomes a critical step to ensure delivery efficiency and quality control.

## 4 Methods for Synthesizing LNPs

**FIGURE 2:**
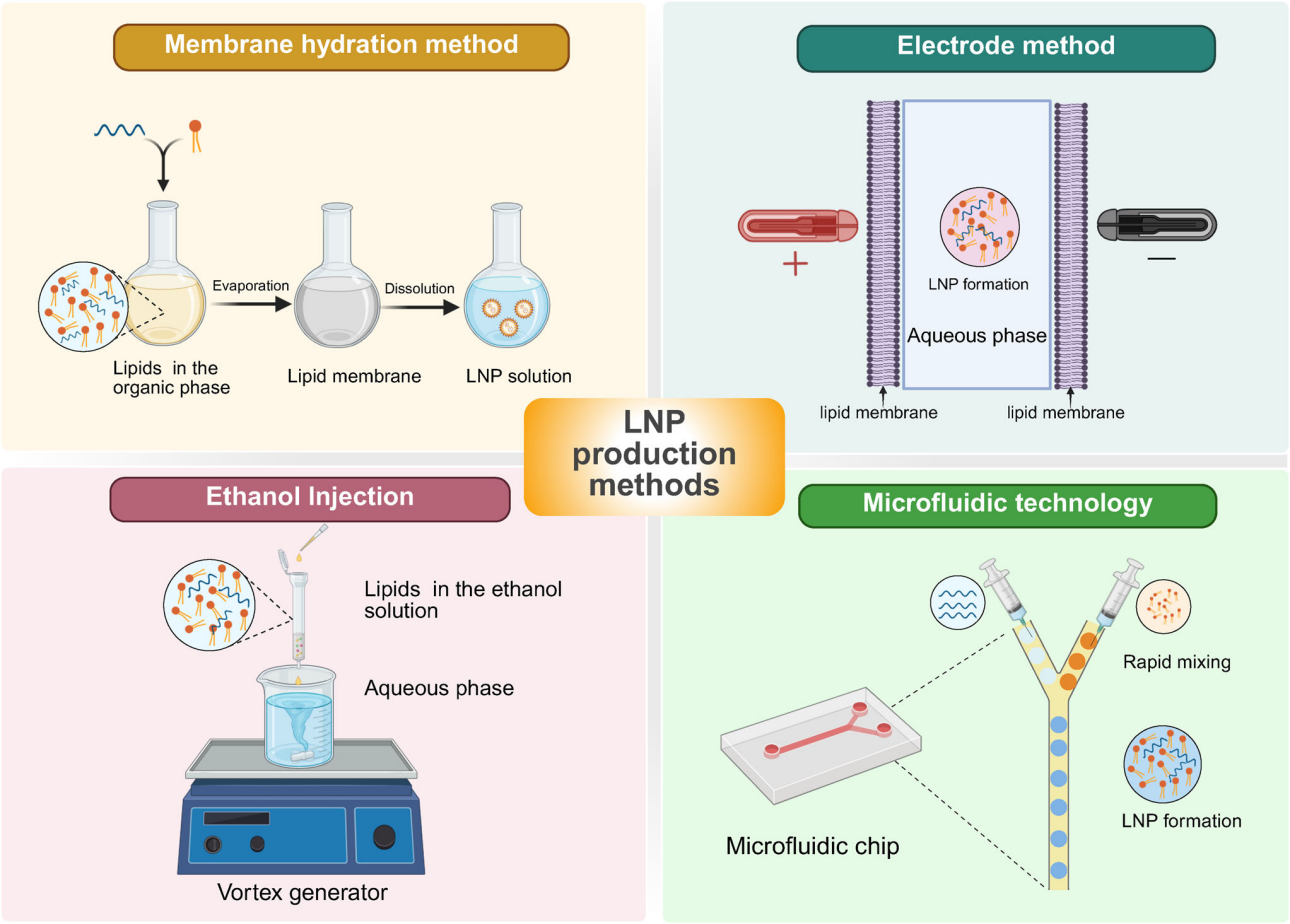
Schematic illustration summarizes four common methods for LNP preparation: membrane hydration, electrode‐based method, ethanol injection, and microfluidic mixing (created in https://BioRender.com).

LNP synthesis relies on electrostatic interactions, van der Waals forces, and hydrophobic effects to form uniformly sized NPs via self‐assembly. The preparation process comprises five key steps: raw material pretreatment, lipid–nucleic acid composite preparation, nanostructure molding, dialysis purification, and terminal sterilization. Selecting an appropriate preparation process is critical. Here, we focus on mainstream methods (Figure 2) and summarize their advantages and disadvantages (Table 1).

**TABLE 1:** Summary of advantages and disadvantages of LNP synthesis methods.

Production method	Advantages	Disadvantages	References
Thin‐film hydration	Multilayer LNPs have small particle size, large volume, wide range of applications, and high alkalinity.	Prone to residual organic solvents	[82, 83]
Electrode method	Larger diameter vesicles, controllable lukewarm conditions	Conductivity dependence, potential electrochemical side effects, low flux	[84]
Ethanol solution	Simple conditions, maneuverability, high mobility	Prone to residual organic solvents	[85, 86]
Microfluidic techniques			
Fluid focusing	Uniform bubble diameter, high dispersion, suitable for high throughput production	Unstable encapsulation rate, difficult to form large vesicles	[87, 88]
Pulse jetting	Controllable vesicle size and dimensions; high encapsulation efficiency and dispersion	Harsh conditions, highly variable	[89]
Solvent emulsion	High sealing speed	Tight control of droplet size	[90, 91]
Ice drop hydration	Simple operation and process	Low encapsulation rate	[92]

The thin‐film hydration method involves mixing phospholipids with organic solvents, such as chloroform, methanol, and ethanol. The organic solvents are then evaporated without hydrodynamics, and the aqueous buffer residue is used to hydrate the organic solvents, leading to spontaneous LNP swelling. This process ultimately forms giant monolayer vesicle [93]. In the electrode method, the lipid membrane is attached to an electrode, followed by the application of an external field to reduce the attraction between the aqueous and lipid phases. This induces unstable membrane bending and the subsequent formation of monolayer lipid vesicles upon hydration under the action of direct and alternating currents [84]. The ethanol injection method, developed by Batzri et al. [94], combines lipid‐carrying monolayer LNPs by naturally fusing the ethanol solution with the aqueous phase [95].

The field of microfluidics is predicated on the principle of unobstructed fluid movement within channels with cross‐sectional dimensions ranging from 5 to 500 µm. The process plant leverages its ability to regulate the self‐assembly of microfluidics by adjusting the flow rate, misflow ratio, composition, and concentration of the fluid to yield LNPs with tunable dimensions [96]. Common microfluidic techniques include fluid focusing, pulse jetting, solvent emulsion, and ice drop hydration. The flow‐focusing technique involves a central stream of ethanol solution containing phospholipids flanked by water streams. As these streams converge to form a microchannel, ethanol diffuses into the aqueous solution, diluting it to a critical concentration that initiates spontaneous lipid assembly into liposomes, with diameters dependent on the fluid's flow rate. Conversely, the pulse‐jet method differs from fluid focusing in that generates a planar lipid membrane vertically from an aqueous n‐decane solution; a jet of liquid ejected from a fine nozzle near the membrane induces deformation and vesicle formation [89].

Tan et al. [97] developed a novel solvent emulsion method utilizing a shear‐focusing droplet production system, where lipids and long‐chain unsaturated lipoteichoic acid constitute the lipid phase, while ethanol and water form the aqueous phase. Upon fusion of these phases, oleic acid rapidly dissolves in the ethanol solution, prompting spontaneous assembly of LNPs, which exhibit long‐term stability [97].

The ice‐drop hydration method typically proceeds as follows. First, sorbitan monooleic acid and stearamine are used as emulsifiers, with a hexane solution as the lipid phase. Monodisperse water‐in‐oil emulsions, with average liquid diameters ranging from 4 to 20 µm are created via microchannels. Second, these emulsions are frozen, and the droplets are separated from the hexane solution. Third, the emulsified droplets undergo a freezing process, leading to their separation from the hexane solution. Next, a hexane solution containing phosphatidylcholine, cholesterol, and stearamine (molar ratio 5:5:1) is used while maintaining droplets in a frozen state. Subsequently, the hexane is evaporated, and the aqueous phase is introduced to the emulsion and droplet mixture. This process forms large vesicles with sizes comparable to the initial emulsion droplets (4–20 µm). Finally, the vesicles are extruded through a polycarbonate membrane to form a monolayer [92].

Microfluidic technology has become the “gold standard” for the industrialized preparation of LNP by virtue of its precise particle size control, efficient production, and experimental reproducibility. It has been demonstrated that this technology overcomes the limitations of traditional methods in terms of particle size control, nucleic acid protection, and process scale‐up. In addition, it provides a technical foundation for the personalized and functionalized design of next‐generation nucleic acid drugs.

The following section will provide a detailed overview of the broad applications of mRNA–LNP vaccines in areas such as infectious diseases and cancer immunotherapy. In the field of disease treatment, the research progress of LNPs in the delivery of proteins, peptides and small molecules has been expanded. Moreover, beyond conventional and rare disease treatment, the LNP platform is being innovatively applied to the immunotherapy of addiction‐related disorders, such as opioid use disorder, thereby expanding the clinical frontiers of mRNA vaccine applications.

## 5 Application of LNPs in Vaccines and Therapeutics

### 5.1 LNPs for mRNA‐Based Infectious Disease Vaccines

LNPs deliver mRNA therapeutics into host cells where the translated target proteins serve as immunogens to elicit immune responses that protect the host from pathogens. Following the success of COVID‐19 mRNA vaccines, this technology has rapidly expanded to target other viral and bacterial pathogens [98]. mRNA–LNP vaccines that target pathogen‐specific antigens are promising for preventing infectious diseases. Table 2 presents the clinical progress of mRNA–LNP‐based vaccines for preventing infectious diseases.

**TABLE 2:** LNP‐based vaccines and therapeutic drugs in clinical trials or approved for marketing.

Intervention	Condition	Load	LNP composition	Route	Sponsor	Trial	Phase
Infectious diseases							
BNT162b2	COVID‐19	Linear mRNA	ALC‐0315:ALC‐0159:DSPC:cholesterol (46.3:1.6:9.2:42.7)	IM	Pfizer/BioNTech	Approved for marketing (US FDA) (2021.8.23)	/
mRNA‐1273	COVID‐19	Linear mRNA	SM‐102:PEG2000‐DMG:DSPC:cholesterol (50:1.5:10:38.5)	IM	ModernaTX, Inc.	Approved for marketing (US FDA) (2022.1.31)	/
ARCT‐154	COVID‐19	sa‐mRNA	ATX‐126:PEG2000‐DMG:DSPC:cholesterol (50:7:40:3)	IM	CSL/Arcturus Therapeutics	Approved for marketing (MHLW) (2023.11.18)	/
mRNA‐1010	Influenza	Linear mRNA	SM‐102:PEG2000‐DMG:DSPC:cholesterol	IM	ModernaTX, Inc.	NCT05827978	III (Completed)
H1ssF‐3928	Influenza	Linear mRNA	Unpublished	IM	NIAID	NCT05755620	I (Active, not recruiting)
ARCT‐2138	Influenza	sa‐mRNA	Unpublished	IM	Arcturus Therapeutics, Inc.	NCT06125691	I (Active, not recruiting)
H3mRNA–LNP	Influenza	Linear mRNA	Unpublished	IM	Sanofi Pasteur	NCT05829356	I (Completed)
Influenza hemagglutinin mRNA vaccine	Influenza	Linear mRNA	Unpublished	IM	Sanofi Pasteur	NCT06118151	I (Completed)
mRNA NA vaccine	Influenza	Linear mRNA	Unpublished	IM	Sanofi Pasteur	NCT05426174	I (Completed)
MRT‐5413	Influenza	Linear mRNA	Unpublished	IM	Sanofi Pasteur	NCT05650554	I/II (Completed)
mRNA‐1345	RSV	Linear mRNA	SM‐102:PEG2000‐DMG:DSPC:cholesterol	IM	ModernaTX, Inc.	Approved for marketing (US FDA) (2024.5.31)	/
JCXH‐108	RSV	Linear mRNA	Unpublished	IM	Immorna Biotherapeutics, Inc.	NCT06564194	I (Active, not recruiting)
IN006	RSV	Linear mRNA	Unpublished	IM	Shenzhen Shenxin Biotechnology Co., Ltd	NCT06645665	I (Recruiting)
CL‐0059/CL‐0137	RSV	Linear mRNA	Unpublished	IM	Sanofi Pasteur,	NCT05639894	I/IIa (Completed)
IN001	Herpes Zoster	Linear mRNA	Unpublished	IM	Shenzhen Shenxin Biotechnology Co., Ltd	NCT06375512	I (Active, not recruiting)
mRNA‐1647	Cytomegalo virus	Linear mRNA	Unpublished	IM	ModernaTX, Inc.	NCT05085366	III (Active, not recruiting)
mRNA‐1644/mRNA‐1644v2‐Core	HIV	Linear mRNA	Unpublished	IM	International AIDS Vaccine Initiative	NCT05001373	I (Active, not recruiting)
mRNA‐1325	Zika virus	Linear mRNA	Unpublished	IM	ModernaTX, Inc.	NCT03014089	I (Completed)
mRNA‐1893	Zika virus	Linear mRNA	Unpublished	IM	ModernaTX, Inc.	NCT04064905	I (Completed)
mRNA‐1215	Nipah virus	Linear mRNA	Unpublished	IM	NIAID	NCT05398796	I (Completed)
mRNA‐1944	Chikungunya Virus	Linear mRNA	Unpublished	IM	ModernaTX, Inc.	NCT03829384	I (Completed)
CV7202	Rabies	Linear mRNA	Unpublished	IM	CureVac	NCT03713086	I (Completed)
BNT164a1	Tuberculosis	Linear mRNA	Unpublished	IM	BioNTech SE	NCT05537038	I (Active, not recruiting)
BNT164b1	Tuberculosis	Linear mRNA	Unpublished	IM	BioNTech SE	NCT05547464	IIa (Recruiting)
mRNA‐1975/mRNA‐1982	Lyme Disease	Linear mRNA	Unpublished	IM	ModernaTX, Inc.	NCT05975099	II (Active, not recruiting)
Cancer therapy							
mRNA‐5671	KRAS mutant advanced, metastatic non‐small cell lung cancer, colorectal cancer, pancreatic adenocarcinoma	Linear mRNA	Unpublished	IM	ModernaTX, Inc.###Merck Sharp & Dohme LLC	NCT03948763	I (Terminated)
mRNA‐4157	Resected solid tumors	Linear mRNA	Unpublished	IM	ModernaTX, Inc.	NCT03313778	I (Recruiting)
BNT131 (SAR441000)	Advanced solid tumors	Linear mRNA	Unpublished	Intratumoral injection	Sanofi	NCT03871348	I (Terminated)
MEDI1191	Advanced solid tumors	Linear mRNA	Unpublished	Intratumoral injection	MedImmune LLC	NCT03946800	I (Completed)
ABOD2011	Advanced solid tumors	Linear mRNA	Unpublished	Intratumoral injection	Cancer Institute and Hospital, Chinese Academy of Medical Sciences	NCT05392699	I (Recruiting)
INT‐1B3	Advanced solid tumors	ncRNA	Unpublished	IV	InteRNA	NCT04675996	I (Terminated)
mRNA‐4157	Advanced melanoma	Linear mRNA	Unpublished	IM	ModernaTX, Inc.	NCT03897881	II (Recruiting)
BNT111	Advanced melanoma	Linear mRNA	Unpublished	IV	BioNTech SE	NCT04526899	II (Active, not recruiting)
BNT122 (RO7198457)	Advanced melanoma	Linear mRNA	Unpublished	IV	Genentech, Inc.	NCT03815058	II (Completed)
mRNA‐2752	Advanced malignancies	Linear mRNA	Unpublished	Intratumoral injection	ModernaTX, Inc.	NCT03739931	I (Active, not recruiting)
mRNA‐2416	Advanced malignancies	Linear mRNA	Unpublished	Intratumoral injection	ModernaTX, Inc.	NCT03323398	I/II (Terminated)
EBV mRNA vaccine	Refractory malignant tumors	Linear mRNA	Unpublished	IM	West China Hospital	NCT05714748	I (Active, not recruiting)
CV09050101 (CVGBM)	Glioblastoma/astrocytoma	Linear mRNA	Unpublished	IM	CureVac	NCT05938387	I (Active, not recruiting)
CV‐8102	Advanced melanoma cSCC hnSCC ACC	Linear mRNA	Unpublished	Intratumoral injection	CureVac	NCT03291002	I
HBV mRNA vaccine	Hepatocellular carcinoma	Linear mRNA	Unpublished	IM	West China Hospital	NCT05738447	I
DCR‐MYC	Hepatocellular carcinoma	siRNA	Unpublished	IV	Dicerna Pharmaceuticals	NCT02314052	Ib/II (Terminated)
OTX‐2002	Hepatocellular carcinoma	Linear mRNA	Unpublished	IV	Omega Therapeutics	NCT05497453	I/II (Terminated)
TKM‐080301	Liver cancer	siRNA	Unpublished	IV	NCI	NCT01437007	I (Completed)
BNT112	Prostate cancer	Linear mRNA	Unpublished	IV	BioNTech SE	NCT04382898	II (Terminated)
BNT113	Form of head and neck cancer	Linear mRNA	Unpublished	IV	BioNTech SE	NCT04534205	II/III (Recruiting)
BI‐1361849	Non‐small cell lung cancer	Linear mRNA	Unpublished	ID	Ludwig Institute for Cancer Research	NCT03164772	I/II (Completed)
Rare diseases							
Onpattro	hATTR	siRNA	DLin‐MC3‐DMA:PEG2000‐DMG:DSPC:cholesterol (50:1.5:10:38.5)	IV	Alnylam	Approved for marketing (US FDA) (2018.8.10)	/
NTLA‐2001	ATTR‐CM	Cas9mRNA/sgRNA–TTR	Unpublished	IV	Intellia Therapeutics	NCT06128629	III (Recruiting)
NTLA‐2001	ATTR‐CM and ATTRv‐PN	Cas9mRNA/sgRNA–TTR	Unpublished	IV	Intellia Therapeutic	NCT04601051	I (Active, not recruiting)
mRNA‐3927	Propionic acidemia	Linear mRNA	SM‐86:0L‐5:DSPC:cholesterol	IV	ModernaTX, Inc.	NCT04159103	I/II (Recruiting)
mRNA‐3705	Methylmalonic acidemia	Linear mRNA	SM‐86:0L‐5:DSPC:cholesterol	IV	ModernaTX, Inc.	NCT04899310	I/II (Recruiting)
mRNA‐3210	Phenylketonuria	Linear mRNA	SM‐86:0L‐5:DSPC:cholesterol	IV	ModernaTX, Inc.	NCT06147856	I/II (Withdrawn)
ARCT‐810	Ornithine transcarbamylase deficiency	Linear mRNA	ATX–Lipid:PEG2000‐DMG:DSPC:cholesterol	IV	Arcturus Therapeutics, Inc.	NCT06488313	II (Recruiting)
MRT‐5201	Ornithine transcarbamylase deficiency	Linear mRNA	Unpublished	IV	Translate Bio, Inc.	NCT03767270	I/II (Withdrawn)
mRNA‐3745	Glycogen storage disease type 1a	Linear mRNA	Unpublished	IV	ModernaTX, Inc.	NCT05095727	II (Recruiting)
UX053	Glycogen storage disease type III	Linear mRNA	Unpublished	IV	Ultragenyx Pharmaceutical Inc	NCT04990388	I/II (Terminated)
VX‐522	Cystic fibrosis	Linear mRNA	Unpublished	Oral inhalation	Vertex Pharmaceuticals Inc.	NCT05668741	II (Recruiting)
MRT‐5005	Cystic fibrosis	Linear mRNA	Unpublished	Nebulization	Translate Bio, Inc.	NCT03375047	I/II
ARCT‐032	Cystic fibrosis	Linear mRNA	Unpublished	Nebulization	Arcturus Therapeutics, Inc.	NCT06747858	II (Recruiting)
mRNA‐6231	Autoimmune disease	Linear mRNA	Unpublished	SC	ModernaTX, Inc.	NCT04916431	I (Completed)
mRNA‐0184	Cardiac failure	Linear mRNA	Unpublished	IV	ModernaTX, Inc.	NCT06243770	I (Recruiting)
NTLA‐2002	Hereditary angioedema	Cas9mRNA/sgRNA–kallikrein	Unpublished	IV	Intellia Therapeutics	NCT06634420	III (Active, not recruiting)

*Note*: Information was obtained from clinicaltrials.gov.

Abbreviations: ACC, adenoid cystic carcinoma; AIDS, acquired immune deficiency syndrome; ATTR‐CM, transthyretin amyloidosis‐related cardiomyopathy; ATTRv‐PN, hereditary transthyretin amyloidosis with polyneuropathy; Cas9, clustered regularly interspaced short palindromic repeats‐associated protein 9; COVID‐19, coronavirus disease 2019; cSCC, squamous cell carcinoma of the skin; CSL, Commonwealth Serum Laboratories; DSPC, 1,2‐distearoyldistearoyl‐sn‐glycero‐3‐phosphocholine; US FDA, United States Food and Drug Administration; GSK, GlaxoSmithKline; HIV, human immunodeficiency virus; hnSCC, squamous cell carcinoma of the head and neck; hTTR, hereditary transthyretin amyloidosis; IM, intramuscular injection; IV, intravenous injection; MHLW, Ministry of Health, Labour and Welfare; NCI, National Cancer Institute; ncRNA, noncoding RNA; NIAID, National Institute of Allergy and Infectious Diseases; RSV, respiratory syncytial virus; SC, subcutaneous injection; sa‐mRNA, self‐amplifying mRNA; sgRNA, single guide RNA; siRNA, small interfering RNA.

mRNA vaccines offer advantages such as flexible antigen design, rapid production cycles, and the ability to induce humoral and cellular immune responses. Recent advancements have been observed in the development of mRNA vaccines targeting various viral pathogens. For instance, the quadrivalent seasonal mRNA–LNP vaccine candidate, mRNA‐1010, encodes four full‐length hemagglutinins. Phase II clinical trials have demonstrated that mRNA‐1010 elicits higher hemagglutination inhibition titers against influenza A strains and comparable titers against influenza B strains. However, preliminary Phase III results indicate a higher incidence of adverse events associated with mRNA‐1010 [99, 100]. mRNA‐1345, approved by the US FDA for preventing respiratory syncytial virus (RSV) infection, is Moderna's second approved product and is currently undergoing Phase I trials in children aged 5–24 months (NCT05743881) and RSV‐seropositive children aged 12–59 months (NCT04528719). In another example, mRNA‐1653 encodes the full‐length membrane‐bound fusion proteins of human metapneumovirus and parainfluenza virus. Results from the Phase I clinical trial (NCT03392389) indicate that intramuscular (IM) administration induces robust neutralizing antibody titers, suggesting the potential to prevent infections by both pathogens [101]. In response to the 2022 monkeypox virus (MPXV) outbreak, mRNA‐1769, encoding four conserved MPXV surface proteins (M1, A29, A35, and B6), exhibited protective efficacy in murine models against vaccinia virus (VACV) and is now in Phase I trials (NCT05995275) [102]. BNT166a, a quadrivalent vaccine encoding M1, A35, B6, and H3 antigens, and its trivalent counterpart BNT166c (excluding H3) both confer protection in mice against VACV, MPXV Clade I and Clade IIb infections. BNT166a has been authorized for Phase I clinical evaluation (NCT05988203) [103]. Tian et al. [104] developed a thermostable ionizable LNP (iLNP) platform encapsulating mRNA encoding the MPXV A29L antigen, which elicited potent cross‐neutralizing immune responses against both vaccinia and monkeypox live viruses. The CV7202 rabies vaccine induced high antibody titers in healthy individuals during a Phase I trial (NCT03713086) [105]. The mRNA‐1647 vaccine for human cytomegalovirus (HCMV), encoding glycoprotein B and the pentameric complex, has demonstrated durable and specific antibody responses in seronegative and seropositive individuals [106]. The BNT163 trivalent HSV‐2 vaccine candidate, encoding gD2, gC2, and gE2, is currently in a Phase I clinical trial (NCT05432583) to evaluate dose escalation, immunogenicity, and safety. Last, two clinical vaccine candidates for varicella‐zoster virus—mRNA‐1468 (encoding glycoprotein E, NCT05701800) and BNT167 (encoding glycoprotein E, NCT05703607)—are both in Phase I clinical development [98].

The prevalence of antimicrobial resistance among human bacterial pathogens underscores the need for effective antibacterial vaccines. However, developing vaccines against bacteria faces greater challenges than viruses due to the larger and more complex bacterial genomes, which present a broader range of potential vaccine targets, including nonprotein antigens currently inaccessible to mRNA vaccine platforms. Moreover, while broad‐spectrum protection against viruses is often associated with high levels of neutralizing antibodies, targeting intracellular bacteria typically requires robust CD8⁺ T cell responses. BNT164a1/BNT164b1, developed by BioNTech, is currently the only multiantigen mRNA vaccine candidate for tuberculosis that has entered clinical trials. The BNT164 vaccine comprises antigens expressed during acute, reactivation, and latent stages of infection: Ag85A (Δ1–41), ESAT‐6, M72, VapB47, Hrp1, RpfA, RpfD, and HbhA. Phase I/II trials began in April 2023 in Germany (NCT05537038) and South Africa (NCT05547464) to evaluate dosage and immunogenicity in HIV‐negative and HIV‐positive individuals, with completion anticipated in 2027 [107]. Another promising candidate, mRNA^CV2^, developed by the University of Sydney, encodes a CysD/Ag85B fusion protein. Preclinical studies have demonstrated its immunogenicity, inducing a Th1‐biased CD4⁺ T cell response and markedly reducing *Mycobacterium tuberculosis* burden in the lungs of infected mice. Recent data suggest it may also enhance the immunogenicity and long‐term protective efficacy of Bacillus Calmette–Guérin vaccination [108]. The mRNA–LNP vaccine candidate mRNA‐1982, which target Lyme disease by encoding the outer surface protein A (OspA) of *Borrelia* spp., is currently undergoing clinical evaluation (NCT05975099). Additionally, researchers at the Israel Institute for Biological Research have developed a bivalent mRNA vaccine that encodes the LcrV and F1 antigens and has demonstrated strong immunogenicity and protective efficacy in preclinical models of pneumonic plague. This vaccine notably induces neutralizing antibodies and robust cellular immunity, thereby providing a dual protection mechanism [109].

Since the COVID‐19 pandemic, mRNA vaccine technology has developed rapidly to combat various viral and bacterial pathogens. With flexible antigen designs, short manufacturing cycles, and the ability to induce potent humoral and cellular immune responses, mRNA vaccines show considerable promise. Several candidates targeting influenza, RSV, CMV, MPXV, HSV, and varicella have advanced to clinical trials with favorable outcomes. However, mRNA vaccines against bacterial pathogens face challenges due to antigenic complexity and the presence of nonprotein targets.

Future advancements in mRNA vaccines are expected to improve mucosal delivery, multivalent design, chronic infectious disease prevention, and the targeting of antimicrobial‐resistant bacteria—ushering in an era of precision, personalization, and broad‐spectrum protection.

### 5.2 LNPs in Cancer Immunotherapy and mRNA‐Based Cell Therapy

Originally developed for the prevention of infectious diseases, mRNA–LNP vaccines have increasingly been applied in immunotherapy due to their protein expression capabilities. At Ruijin Hospital, researchers formulated LNPs using 1,2‐dioleoyl‐3‐trimethylammonium‐propane (DOTAP), phosphatidylcholine, DMG‐PEG 2000, cholesterol, and palmitic acid through self‐assembly, with an optimized formulation ratio of 47.5:10:1.5:36:5 to encapsulate VEGF‐C mRNA. In vitro transfection of H9c2, AC16, and HEK293T cells demonstrated specific expression of the encoded protein. In a C57BL/6 mouse model of myocardial infarction, intramyocardial delivery of VEGF‐C mRNA–LNPs at multiple injection sites promoted lymphangiogenesis, improved cardiac function, and decreased inflammation [110]. These favorable therapeutic outcomes were largely attributed to palmitic acid, which provides carboxyl groups that bind the extracellular matrix and prolong LNP retention within myocardial tissue, enhancing transfection efficiency. Table 2 presents the clinical progress in mRNA–LNP tumor therapeutic vaccines.

mRNA vaccines are being explored as a platform for cancer vaccine development. These vaccines involve the in vitro synthesis of mRNA sequences encoding tumor antigens, which are delivered into host cells to be translated into proteins, thus activating the immune system. Research on mRNA cancer vaccines is primarily focused on two categories of antigens: TAAs and tumor‐specific antigens (TSAs) [111]. Initial efforts concentrated on TAAs, but their broad expression and associated immune tolerance limited therapeutic efficacy. In contrast, personalized vaccines based on patient‐specific neoantigens (TSAs) have demonstrated enhanced specificity and are now a major focus in the field. Once delivered, the mRNA is translated into antigenic proteins in the cytoplasm of antigen‐presenting cells (APCs) and activates Toll‐like receptor (TLR7) and TLR8 signaling pathways. The resulting peptides are presented by MHC class I molecules to activate CD8^+^ T cells, enabling targeted tumor cell killing. Researchers at the University of Pennsylvania found that codelivering IL‐12‐encoding mRNA with antigen‐coding mRNA–LNPs boosts CD8^+^ T cell quantity, function, and efficacy, reducing tumor growth in a melanoma mouse model [112]. Oberli et al. [113] utilized microfluidic technology to formulate LNPs (B‐11; composed of ckk‐E12:DOPE:cholesterol:C14‐PEG2000:sodium lauryl sulfate in a 15:26:40.5:2.5:16 ratio) encapsulating mRNA encoding ovalbumin (OVA), tyrosinase (TYP)‐related protein 2, or the gp100 peptide with a position 27 mutation. In mice bearing B16F10 tumors, these vaccines elicit strong antigen‐specific CD8^+^ T cell responses and reduce tumor growth. Adding lipopolysaccharide to the formulation improved antitumor efficacy and prolonged survival [113]. An LNP‐encapsulated personalized mRNA vaccine, mRNA‐4157, encodes 34 neoantigens (29 MHC I and 5 MHC II epitopes). In the KEYNOTE‐942 (Phase IIb) clinical trial (NCT03897881), mRNA‐4157/V940 combined with pembrolizumab reduced recurrence risk by 44% in melanoma patients [114]. Another candidate, Autogene cevumeran (BNT122), encoding 20 TSAs combined with atezolizumab, showed strong CD8^+^ T cell responses and reduced recurrence risk in patients with pancreatic ductal adenocarcinoma (PDAC). Additionally, BNT116, an RNA–lipid complex encoding six tumor antigens (MAGE‐A3, CLDN6, KK‐LC‐1, PRAME, MAGE‐A4, and MAGEC1), demonstrated favorable tolerability and safety in a Phase I trial (NCT05142189) for non‐small cell lung cancer, either alone or combined with cemiplimab. A Phase II trial (NCT05557591) is ongoing, expected to conclude by 2027 [115]. The BNT111 vaccine encodes four TAAs: NY‐ESO‐1, TYP, melanoma‐associated antigen A3 (MAGE‐A3), and transmembrane phosphatase with tensin homology. A Phase I trial (NCT02410733) demonstrated that BNT111 induced strong CD4^+^ and CD8^+^ T cell responses in melanoma patients. Additionally, the ongoing Phase II trial (NCT04526899) combining BNT111 with cemiplimab has shown encouraging therapeutic outcomes [116].

Chimeric antigen receptor T cell (CAR‐T) immunotherapy also exhibits promise in treating certain hematological malignancies, such as diffuse large B‐cell lymphoma, acute lymphoblastic leukemia, and BCMA‐positive multiple myeloma. The primary CAR gene delivery method is viral transduction, accounting for 94% of assessable CAR‐T products, with lentiviral vectors comprising 53.3%. However, the random integration of lentiviral vectors into the host genome may disrupt essential genes or activate oncogenes, potentially leading to insertional mutagenesis [117, 118]. To address these concerns, Chen et al. [119] employed a microfluidic device to encapsulate human CAR mRNA targeting CD19 into LNPs (DLin‐MC3‐DMA:DSPC:DSPE‐PEG2000:cholesterol formulation at 50:10:1.5:38.5). They optimized transfection protocols to deliver mRNA into T cells, with mRNA entering the cytoplasm within 8 h, resulting in robust CAR expression on the T cell surface. Coculturing with CD19‐positive tumor cell lines (Daudi, Raji, and Nalm‐6), mRNA–LNP‐engineered CAR‐T cells exhibited high surface expression of CD107a—a marker transiently expressed during degranulation of cytotoxic T cells upon target cell engagement. Furthermore, in a 3D tumor spheroid model, the engineered CAR‐T cells demonstrated excellent infiltration and cytotoxicity. Meanwhile, in vivo, these cells home to tumor sites in B cell malignancy mouse models, significantly suppressing tumor growth. In addition to ex vivo modification of T cells, Hunter et al. [120] devised an innovative strategy for in vivo CAR‐T cell generation using targeted LNPs (tLNPs) formulated with an ionizable lipid (L829). By conjugating tLNPs with anti‐CD5 or anti‐CD8 antibodies, the system enabled selective delivery of CAR‐encoding mRNA into specific T‐cell subsets while minimizing off‐target accumulation in the liver. In humanized mouse models, administration of tLNPs rapidly produced functional CAR‐T cells within hours, leading to efficient eradication of B cell malignancies and solid tumors, with nearly complete tumor regression. In cynomolgus monkeys, intravenous (IV) administration resulted in profound but reversible B cell depletion and was well tolerated. This approach eliminates the need for ex vivo cell manufacturing and preconditioning chemotherapy, thereby addressing several key limitations of current CAR‐T therapies and underscoring the transformative potential of mRNA–LNP‐based in vivo CAR‐T engineering [120]. mRNA–LNP technology also holds promise for treating solid tumors. The BNT211 platform combines CAR‐T cells targeting the TAA CLDN6 with an mRNA vaccine encoding CLDN6 (termed CARVac), enhancing efficacy in CLDN6‐positive solid tumors. Preclinical solid tumor models and a Phase I clinical trial (NCT04503278) reported good safety, tolerability, and preliminary antitumor activity [121, 122].

mRNA–LNP technology, originally developed for infectious disease vaccines, has rapidly evolved into a versatile therapeutic platform with broad applications in immunotherapy, particularly in oncology and regenerative medicine. The ability of mRNA to encode specific proteins has enabled its use in targeted therapies, such as delivering VEGF‐C mRNA to promote lymphangiogenesis and cardiac repair following myocardial infarction. In cancer immunotherapy, mRNA–LNP vaccines have progressed from encoding TAAs to highly personalized vaccines based on TSAs, demonstrating robust activation of cytotoxic T cell responses and significant clinical benefits in various cancers, including melanoma, PDAC, and non‐small cell lung cancer. Several candidates, such as mRNA‐4157, BNT122, and BNT111, are undergoing clinical trials with promising outcomes in terms of tumor regression and recurrence reduction. Beyond vaccines, mRNA–LNPs are also being explored for engineering CAR‐T cells, offering a nonviral, transient, and potentially safer alternative to conventional gene delivery methods. LNP‐formulated CAR mRNAs have shown strong expression, tumor infiltration, and cytotoxic activity in both hematological and solid tumor models. These advances highlight the expanding therapeutic potential of mRNA–LNPs, supporting their continued development as a transformative modality in precision medicine.

### 5.3 LNPs for Gene Therapy and Genome Editing

siRNA molecules can specifically silence the expression of target genes, and LNPs have proven to be effective carriers for siRNA‐based therapeutics. A notable example is the US FDA‐approved product Onpattro, which has demonstrated clinical efficacy and has since been explored for the treatment of various other diseases. Leveraging the unique advantages of LNP technology platforms, gene therapeutics can be selectively delivered to diseased tissues for targeted intervention.

Zhang et al. [123] utilized microfluidic technology to synthesize targeted LNPs composed of SM‐102, DSPC, cholesterol, DMG‐PEG2000, and DMG‐PEG2000‐Maleimide at a molar ratio of 42:13:43:1.5:0.5. These LNPs were subsequently modified with lactoferrin (Lf), enabling selective delivery to respiratory epithelial cells and neurons in Alzheimer's disease (AD) models. The LNPs efficiently encapsulated siRNA targeting β‐site amyloid precursor protein cleaving enzyme 1 (BACE1). In vivo studies revealed that gene therapy significantly reduced escape latency in mice, improved spatial memory, and thereby showed potential to delay the progression of AD [123]. Hofstraat et al. [124] developed a prototype apolipoprotein‐based NP (aNP) that effectively targeted myeloid cells, hematopoietic stem cells, and progenitor cells in the bone marrow. These aNPs were capable of encapsulating siRNA against lysosome‐associated membrane glycoprotein 1 (Lamp1), offering a novel strategy for the treatment of bone marrow malignancies [124]. Cui et al. [125] synthesized LNPs composed of Dlin‐MC3‐DMA, cholesterol, DSPC, and DMG‐PEG, coencapsulating siRNA targeting epidermal growth factor receptor (EGFR) and gentamicin (GM). Results demonstrated that GM enhanced endosomal escape of siRNA, thereby improving gene silencing efficacy. In murine tumor models, the LNP–siEGFR/GM system achieved an 81% knockdown of EGFR mRNA, significantly inhibited tumor growth, and induced tumor cell apoptosis, indicating its promising potential in cancer therapy [125]. Overall, the use of LNPs for siRNA delivery enables efficient gene silencing for therapeutic purposes. Furthermore, the integration of LNP‐based delivery systems with gene‐editing technologies is rapidly advancing, especially in the context of cancer treatment.

CRISPR/Cas9 in vivo gene‐editing therapy, using mRNA technology and LNPs that coencapsulate Cas9 mRNA and target specific single guide RNAs (sgRNAs), can be applied to various conditions [126]. Intellia Therapeutics’ NTLA‐2001 targets transthyretin (TTR) for treating transthyretin amyloidosis (ATTR). The LNP formulation (LP000001:DSPC:cholesterol:DMG‐PEG2k = 23.6:4.0:8.2:4.2) encapsulates Cas9 mRNA and a TTR‐specific sgRNA. Phase I trial data (NCT04601051) showed NTLA‐2001 significantly reduced plasma TTR protein levels in patients with ATTR, encouraging the development of CRISPR/Cas9‐mRNA‐based therapies for cancer [127]. Dan Peer's team [126] employed an LNP formulation (DLin‐MC3‐DMA:DSPC:cholesterol:DMG‐PEG:DSPE‐PEG = 50:10.5:38:1.4:0.1) to codeliver Cas9 mRNA and sgRNA targeting PLK1. A single intracranial injection into an aggressive orthotopic glioblastoma model achieved approximately ∼70% in vivo gene editing, suppressed tumor growth by 50%, and increased survival by 30%. Meanwhile, Mitchell's team [128] at the University of Pennsylvania synthesized 252 distinct siloxane‐based lipids to enhance mRNA endocytosis and endosomal escape. Their Si5‐N14 LNP formulation, codelivering Cas9 mRNA and a GFP sgRNA, enabled efficient pulmonary delivery and potent gene knockout in Lewis lung carcinoma‐bearing mice [128].

LNPs have emerged as a powerful platform for the delivery of siRNA and CRISPR/Cas9 gene‐editing systems, enabling precise gene silencing and editing in vivo. siRNA‐loaded LNPs offer a promising therapeutic avenue for diverse diseases by specifically downregulating the expression of pathogenic genes. A landmark in this field is Onpattro—the first US FDA‐approved LNP–siRNA therapeutic—which has validated the clinical potential of LNPs and spurred further exploration into neurodegenerative disorders, cancer, and hematological diseases. Advanced engineering of LNPs, such as surface modification with ligands like Lf or apolipoprotein, enables targeted delivery to specific cell types including neurons, respiratory epithelial cells, and hematopoietic cells. Codelivery strategies, such as combining siRNA with GM to enhance endosomal escape, have further improved gene silencing efficacy in cancer models. Beyond RNA interference, LNPs have also become a key vehicle for in vivo CRISPR/Cas9 gene editing. Platforms like NTLA‐2001 demonstrated successful gene knockout in clinical trials for transthyretin amyloidosis, while preclinical studies using LNPs to deliver Cas9 mRNA and sgRNA in glioblastoma and lung cancer models achieved high editing efficiency, tumor suppression, and extended survival. These breakthroughs highlight the expanding role of LNPs in next‐generation gene therapies, particularly for diseases where precision and safety are critical.

### 5.4 LNPs for Protein, Peptide, and Small Molecule Delivery

LNPs were initially developed and optimized for nucleic acid delivery. However, they have recently demonstrated substantial potential for encapsulating and delivering other classes of therapeutics, including proteins, peptides, and small molecules. Their favorable biocompatibility, scalable formulation processes, and ability to enhance drug solubility and stability make LNPs a versatile platform beyond nucleic acid‐based therapies.

The delivery of therapeutic proteins and peptides presents considerable challenges due to their susceptibility to enzymatic degradation, poor membrane permeability, and rapid clearance from the body. LNPs can overcome these limitations by encapsulating proteins and peptides within a protective lipid matrix, thereby extending their circulation time, enhancing cellular uptake, and enabling targeted delivery. Several studies have elucidated the application of LNPs in protein delivery. Proteins are amphoteric macromolecules, and under physiological conditions, most exhibit a net positive charge, behaving as polycations [129]. Meagher et al. [130] replaced the ionizable lipid SM‐102 with 50% of the anionic lipid 1,2‐dimyristoyl‐sn‐glycero‐3‐phospho‐(1′‐rac‐glycerol) sodium salt (DMPG) to formulate LNPs that encapsulated various proteins, including fluorescent proteins (mCherry, EBFP2, and Venus), bovine serum albumin, and monoclonal antibodies (trastuzumab and IgG). The results demonstrated that these LNPs significantly enhanced protein encapsulation efficiency and maintained good stability. Robust intracellular fluorescence signals were detected in A549 and SKBR3 cells, indicating efficient protein delivery. Furthermore, trastuzumab‐loaded LNPs specifically bound to HER2 receptors in HER2‐overexpressing SKBR3 cells, confirming that the antibody retained its functional activity postencapsulation [130]. This study supports the feasibility of intracellular delivery of therapeutic proteins via LNPs and holds promise for expanding protein‐based therapies in cancer, cardiovascular, and metabolic diseases. Further exploration of the influence of various anionic lipids on protein encapsulation efficiency could help optimize LNPs for more effective protein delivery. Mastrobattista et al. [131] designed a five‐component LNP formulation (C12‐200:DOPE:cholesterol:PEG‐DMG:DOTAP = 35:16:46.5:2.5:0.25) to encapsulate ribonucleoprotein complexes consisting of Cas9 protein and sgRNA. In vitro experiments demonstrated gene‐editing activity in HEK293T and HEPA1‐6 cells. Moreover, biodistribution and in vivo gene editing studies in female Ai9 mice following systemic administration revealed that LNPs primarily accumulated in the liver, spleen, and lungs, where they achieved effective gene knockout with minimal toxicity [131].

Drug loading within LNPs typically relies on electrostatic interactions between the therapeutic cargo and lipid components. However, peptides often lack a strong net charge, making it difficult for them to interact effectively with LNPs through conventional electrostatic mechanisms. Instead, peptide backbones are rich in amide bonds, which can act as hydrogen bond donors or acceptors. To address this challenge, the research group led by De Geest [132] developed a novel gallic acid‐derived lipid‐based LNP system. In this design, the ionizable lipid head group was replaced with a 3,4,5‐trihydroxyphenyl moiety to enable hydrogen bonding with peptide molecules. The resulting ionizable lipid, S‐GA‐DOG, was constructed by linking the head group to the hydrophobic alkyl tail via a disulfide bond, allowing for redox‐responsive release. When combined with DSPC, cholesterol, and DMG‐PEG, the resulting LNPs were able to successfully encapsulate therapeutic OVA antigen peptides. These peptide‐loaded LNPs facilitated intracellular delivery and effectively activated CD8⁺ T‐cell immune responses, demonstrating their potential for functional peptide‐based immunotherapies [132]. Gu et al. [133] designed quaternary ammonium‐based ionizable lipids incorporating phenylboronic acid groups to form a glucose‐responsive LNP system. These LNPs were capable of efficiently loading recombinant human insulin along with a glucose‐sensing component. Upon exposure to glucose‐rich environments, such as those mimicking hyperglycemia, the insulin was selectively released. In a type 1 diabetes mouse model, this formulation exhibited prolonged glycemic control. This glucose‐responsive LNP platform holds promise as a novel therapeutic strategy for diabetes treatment [133].

Proteolysis‐targeting chimeras (PROTACs) are capable of selectively degrading target proteins within cells. Chan et al. [134] developed a strategy to deliver PROTAC small molecules by forming electrostatic complexes with LNPs containing a fifth lipid component, DOTAP, thereby achieving efficient cytosolic delivery. Their study demonstrated that the K1–LNP formulation—composed of C12‐200:DOPE:C14PEG‐2000:cholesterol:DOTAP at a molar ratio of 19:19:4:38:20—achieved the highest protein degradation efficiency. This platform enabled rapid and robust intracellular protein degradation, with clearance rates reaching up to 95%. Moreover, it allowed targeting of proteins located in diverse subcellular compartments and offered substrate‐specific, repeatable degradation profiles. The modularity of this approach supports the design of clinically relevant protein degraders for therapeutic purposes [134]. Liang et al. [135] developed a peptide‐modified LNP system functionalized with an Asp–Gly–Arg sequence, enabling the effective codelivery of microRNA‐150 and the antiangiogenic agent quercetin. These LNPs demonstrated selective targeting of endothelial cells during abnormal angiogenesis. In a mouse model, the system showed promising therapeutic potential for age‐related macular degeneration, with no observed retinal toxicity [135]. Van der Meel et al. [136] coencapsulated siRNA targeting the androgen receptor and a lipophilic paclitaxel prodrug derivative within LNPs. Using dual radiolabeling techniques, they quantitatively evaluated the pharmacokinetics and biodistribution of the LNPs following systemic administration in tumor‐bearing mice. This codelivery platform highlights a novel approach for the development of combination therapies, facilitating simultaneous delivery of gene silencers and chemotherapeutic agents [136].

The versatility of LNPs as a delivery platform has extended far beyond nucleic acid therapeutics, enabling the efficient encapsulation and targeted delivery of diverse therapeutic agents—including proteins, peptides, small molecules, and combination regimens such as PROTACs, siRNA, and chemotherapeutics—thereby offering innovative strategies for the treatment of cancer, metabolic disorders, and other complex diseases.

### 5.5 LNPs for Rare Diseases and Emerging Applications

LNPs play a crucial role in mRNA replacement therapy for inherited metabolic disorders. Khoja et al. [137] successfully encapsulated mRNA encoding arginase within LNPs, and in arginase‐deficient knockout mouse models, the therapy effectively treated arginase deficiency, prevented associated protein malnutrition, and restored normal oligodendrocyte function. Sahay and colleagues [138] developed LNPs using PEG–lipids modified with carboxylic lipids to encapsulate mRNA encoding recombinases, achieving efficient transfection of photoreceptor cells. Moreover, by coencapsulating Cas9 mRNA and sgRNA, gene editing was successfully performed in the retina, correcting blindness‐causing mutations and demonstrating therapeutic potential for inherited retinal diseases [138]. A preclinical study by CureVac demonstrated that LNPs encapsulating mRNA encoding phenylalanine hydroxylase, when delivered intravenously, rescued metabolic defects in phenylalanine hydroxylase‐deficient mouse models [139]. mRNA‐3927, a therapeutic candidate encoding the alpha (PCCA) and beta (PCCB) subunits of propionyl‐CoA carboxylase, delivered via LNPs, restored hepatic metabolic function and showed potential in treating propionic acidemia [140]. In another preclinical study led by Moderna, LNPs encapsulating mRNA encoding argininosuccinate lyase were intravenously administered in a mouse model of argininosuccinic aciduria (ASA). The results showed an effective blockade of glutathione metabolism, confirming the therapeutic potential of mRNA‐based intervention [141]. Translate Bio developed LNP‐formulated mRNA encoding α‐galactosidase A, demonstrating favorable therapeutic outcomes in Fabry disease mouse models [142]. Moderna also investigated LNPs encapsulating mRNA encoding porphobilinogen deaminase (PBGD), and systemic administration in mouse models significantly reduced the levels of δ‐aminolevulinic acid and porphobilinogen, providing the first in vivo evidence that mRNA‐based therapy can effectively address PBGD deficiency [143]. Furthermore, the Moderna team designed LNPs encapsulating mRNAs encoding key functional proteins and enzymes, showing therapeutic potential for treating Crigler–Najjar syndrome and glycogen storage disease [144].

Researchers at the University of Tübingen employed chitosan‐modified PLGA NPs to deliver chemically modified cystic fibrosis transmembrane conductance regulator (CFTR) mRNA. This approach led to abundant expression of CFTR protein in the lungs and significantly improved pulmonary function and pathological features in CFTR‐deficient mice, effectively treating cystic fibrosis [145]. In a collaborative study by Ludwig Maximilian University of Munich and Hannover Medical School, a class of self‐assembled peptide–PEI NPs (T704) were developed to deliver chemically modified CFTR mRNA. The mRNA was efficiently expressed both in vitro and in vivo. Further experiments demonstrated that T704 NPs could also deliver a Sleeping Beauty transposon system to insert the CFTR gene, thereby enabling long‐term correction of CFTR deficiency with excellent safety and minimal toxicity in both cellular and animal models [146]. In another study, IV administration of LNPs encapsulating mRNA encoding SLC25A13 successfully led to hepatic expression of functional aspartate/glutamate carrier protein in mice, correcting metabolic abnormalities. Treated animals also showed improvements in physical development and behavioral deficits, suggesting therapeutic efficacy in citrin deficiency [147]. A study conducted at Ludwig Maximilian University of Munich was the first to demonstrate that repeated administration of chemically modified mRNA could restore the expression of pulmonary surfactant protein B (SP‐B) and prevent respiratory failure in mice. Delivery of SP‐B mRNA achieved over 70% of normal SP‐B protein levels in the lungs, maintaining both pulmonary structure and function [148]. Building on this, researchers at the University of Tübingen developed a lung‐targeted LNP formulation modified with a specific targeting peptide for SP‐B mRNA delivery. Upon intratracheal administration, the system partially restored SP‐B mRNA and protein expression in pulmonary cells and improved survival in SP‐B‐deficient mice [149]. This study highlights the potential of airway‐delivered, lung‐targeted LNP–mRNA systems for treating SP‐B deficiency.

Most inherited metabolic disorders currently lack effective therapeutic options. For certain conditions characterized by high incidence, significant clinical severity, and favorable outcomes when diagnosed and treated early, the combination of mRNA–LNP therapy with existing clinical interventions offers promising potential. This approach may provide a novel therapeutic avenue for overcoming rare genetic diseases. Recent studies have reported encouraging progress in the application of LNPs within these emerging areas.

Opioids are among the most utilized drugs for pain relief, with common examples including morphine, fentanyl, oxycodone, heroin, and methadone. These small‐molecule drugs interact with opioid receptors, reducing neuronal excitability and impairing the release of nociceptive neurotransmitters, thereby alleviating pain. Furthermore, opioids can induce euphoria, contributing to their high potential for abuse and addiction, frequently culminating in respiratory depression and death [150].

Current pharmacological interventions primarily rely on opioid receptor agonists and antagonists. While these therapies offer some efficacy, they are insufficient for individuals facing long‐term opioid dependence. Antiopioid immunotherapy is emerging as a promising alternative, aiming to sequester opioids outside the receptor space to retain analgesic effects while minimizing adverse side effects [151]. De Geest et al. [152] developed a NP vaccine by noncovalently assembling a fentanyl hapten, CD4⁺ T‐helper peptide epitopes, and a TLR7/8 agonist within LNPs. This formulation effectively induces the production of fentanyl‐specific IgG antibodies and subclasses, which selectively bind opioid molecules and restrict their distribution to the bloodstream and peripheral tissues, preventing interaction with central nervous system receptors. In preclinical mouse models, this approach significantly mitigates the effects of opioid overdose [152]. Cao's team [153] developed a morphine‐encapsulating LNP formulation (Tet1–LNP) that utilizes peptide recognition—specifically, Tet1's binding to trisialoganglioside (GT1b) receptors—to selectively activate peripheral opioid receptors in dorsal root ganglia. This strategy markedly reduces side effects associated with central opioid receptor activation and demonstrates potent and sustained analgesic effects with favorable safety in animal models [153].

Recently, LNP‐based immunotherapy against opioids has emerged as a novel approach to treat addiction, offering therapeutic opportunities for chronic pain management and addiction intervention. The application of LNPs across various clinical settings relies heavily on the high‐quality structural design of mRNA. To further enhance vaccine efficacy, strategies are employed to optimize the mRNA molecule, enabling better synergistic interaction with LNPs.

## 6 Engineering Strategies for mRNA and LNPs Compatibility

The therapeutic efficacy of mRNA‐based vaccines and therapeutics depends not only on the effective delivery of the mRNA payload, but also on the molecular compatibility and co‐optimization between the mRNA and the LNPs carrier. Although LNP technologies have made significant advances in promoting mRNA delivery, accumulating evidence indicates that rational design of the mRNA itself can synergize with LNPs to enhance formulation stability and delivery efficiency.

### 6.1 mRNA Length and Structural Features

The physical properties of mRNA, particularly its length and secondary structure, directly affect its encapsulation efficiency within LNPs. mRNA molecules of moderate length (typically 1–3 kilobases) are more readily encapsulated due to their favorable flexibility and reduced steric hindrance during the self‐assembly of LNPs [154]. In contrast, longer mRNA transcripts—especially those rich in secondary structures, may interfere with NP formation, leading to heterogeneous particle sizes and decreased encapsulation efficiency. Complex secondary structures such as excessive stem‐loops increase molecular rigidity, occupy more space, and hinder lipid interaction with the phosphate backbone, thereby compromising encapsulation. Codon optimization and RNA folding algorithms (e.g., LinearDesign and mRNAid) have been utilized to reduce secondary structure formation, resulting in improved encapsulation [155]. Selecting synonymous codons that are less prone to forming stable local structures can help minimize folding complexity. High GC content tends to stabilize secondary structures; therefore, appropriate adjustment of GC content is essential to balance RNA stability with structural manageability. These strategies work synergistically with lipid components in LNPs to enhance the efficiency of mRNA encapsulation.

### 6.2 Chemical Modification of Nucleosides

Chemical modification of nucleosides is a foundational approach to improve the pharmacological properties of synthetic mRNA by increasing stability, translation efficiency, and immune tolerance. Unmodified mRNA contains immunogenic motifs that trigger PRRs such as TLR3, TLR7/8, RIG‐I, and MDA5, leading to type I interferon responses and inflammation [156]. Incorporating modified nucleosides reduces this immune activation while maintaining or enhancing protein expression. N1‐methyl‐pseudouridine (m^1^Ψ) is the most widely used modification in clinical mRNA vaccines. It suppresses PRR activation, reduces IFN‐α and TNF‐α production, and enhances translational efficiency and molecular stability. Pseudouridine (Ψ) similarly enhances base stacking and dampens innate immune recognition, though with slightly lower translational benefit compared with m^1^Ψ [157]. 5‐Methylcytidine, especially when used with m^1^Ψ, synergistically promotes translation and reduces immunogenicity by improving resistance to endonuclease degradation [158]. N6‐methyladenosine, a natural epitranscriptomic mark, has been explored for translational regulation and immune evasion [159]. Most current designs favor global substitution of uridine with m^1^Ψ or Ψ. Site‐specific incorporation, enabled by enzymatic or solid‐phase synthesis techniques, is being explored for applications requiring precise structural control. However, excessive or mispositioned modifications may impair RNA folding and reduce expression. Thus, modification strategies must balance immune evasion, translation efficiency, and protein integrity.

Incorporation of modified nucleotides, such as m^1^Ψ, into mRNA sequences not only reduces activation of innate immune responses but also enhances mRNA stability and improves its physicochemical compatibility with LNPs. These nucleotide modifications alter the charge distribution along the mRNA backbone, which is inherently highly negatively charged and poses challenges for efficient encapsulation and cellular uptake [160]. Ionizable lipids within LNPs carry positive charges at low pH, enabling electrostatic complexation with the mRNA's phosphate backbone. Rational mRNA design should aim to minimize immunostimulatory motifs—such as U‐ or A‐rich regions—and avoid excessive charge clustering, thereby reducing molecular rigidity [161]. Such strategies facilitate more efficient incorporation of mRNA into LNPs, promote uniform NP formation, enhance encapsulation efficiency, and reduce innate immune recognition.

Once the target protein sequence is determined, optimization of the mRNA sequence and structural features can be performed to improve its encapsulation efficiency within LNPs. In addition, rigorous purification methods such as high‐performance liquid chromatography (HPLC) or tangential flow filtration (TFF) are employed to remove impurities generated during in vitro transcription [162]. Among these, double‐stranded RNA (dsRNA) contaminants are particularly problematic due to their higher rigidity, larger size, and distinct charge density compared with single‐stranded mRNA. dsRNA can compete with the target mRNA for lipid‐binding sites, severely disrupting the encapsulation process and reducing the efficiency of mRNA loading into LNPs [163]. Ensuring uniform mRNA transcript length and minimizing molecular heterogeneity also contributes to achieving more consistent and homogeneous LNP formulations. While the design of the mRNA molecule itself is undoubtedly important, the delivery efficiency also depends on the performance of the carrier system. To support the clinical translation of LNPs technology, it is essential to elaborate on the key strategies for optimizing LNPs design, including targeted drug delivery, enhancement of endosomal escape for efficient cargo release, and mitigation of administration‐related adverse effects. These approaches provide a theoretical foundation for expanding the therapeutic applications of LNPs.

## 7 Optimizing the Design of LNPs

COVID‐19 mRNA vaccines have increased the use of LNPs as delivery media. Despite their promise, LNPs face several challenges, including poor targeting, insufficient endosomal escape, adverse reactions, and degradation byproducts that form adducts with mRNA molecules, diminishing their expression. This section offers theoretical support for the rational design of next‐generation LNPs.

### 7.1 LNP Targeting

**FIGURE 3:**
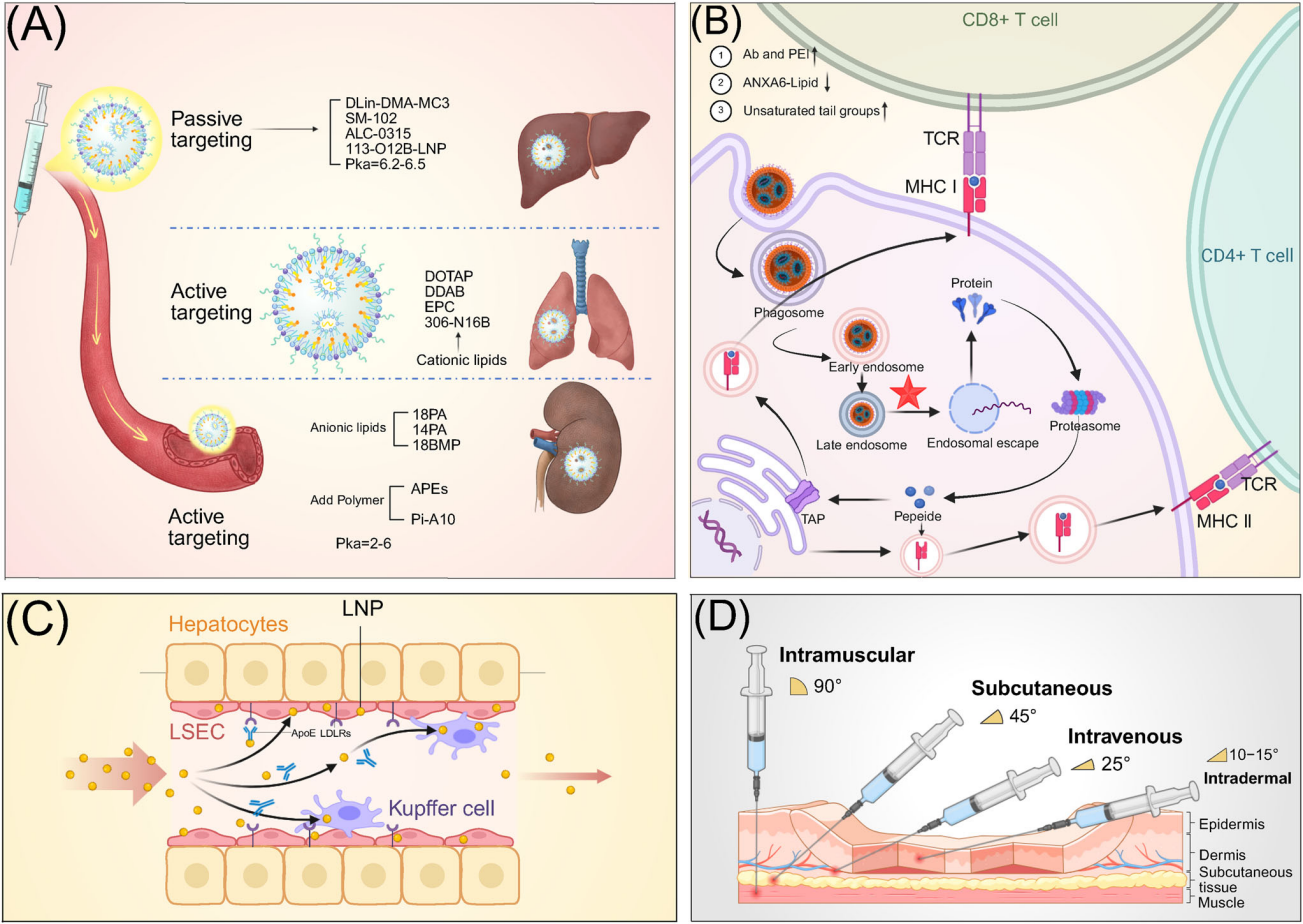
The optimization strategies for enhancing LNP targeting and endosomal escape are summarized as follows: panel (A) illustrates organ‐selective delivery profiles of various LNP formulations. Conventional ionizable lipids with p*K*_a_ values ranging from 6.2 to 6.5 (e.g., DLin‐DMA, MC3, SM‐102, ALC‐0315, and 113‐O12B) exhibit liver tropism. Permanently ionizable lipids (e.g., DOTAP, DDAB, EPC, and 306‐N16B) favor lung targeting, while anionic lipids (e.g., 18PA, 14PA, and 18BMP) and polymer modifications (e.g., APEs and Pi‐A10) are associated with spleen accumulation. Panel (B) depicts key mechanisms of endosomal escape that enhance both CD4⁺ and CD8⁺ T cell responses. Strategies include conjugating LNPs with antibodies or PEI polymers, inhibiting the interaction between LNPs and ANXA6, and employing ionizable lipids with unsaturated hydrophobic tails, which significantly improve endosomal escape efficiency and thereby enhance antigen expression and immune activation. Panel (C) shows that LNPs can bind to ApoE, which facilitates recognition by LDLRs on hepatic endothelial cells, contributing to hepatic accumulation—a typical feature of traditional LNPs. Panel (D) outlines injection sites for different administration routes (intramuscular, subcutaneous, intravenous, and intradermal), which influence how LNP‐based vaccines enter the body and determine the type and strength of immune responses elicited (created in https://BioRender.com).

LNPs ten to accumulate in the liver when administered intravenously or intramuscularly, which impedes the efficacy of mRNA vaccines [164]. This is due to LNPs adsorbing ApoE in the plasma, binding LDLRs, and entering liver cells [165]. Although this process helps target drugs to the liver, it reduces their systemic efficacy. Consequently, researchers have sought to modify LNPs to enhance organ targeting by altering their surface charge, composition, structure, and size [166, 167]. This field has garnered considerable global interest, advancing our understanding of LNP adaptation to immune organs [168, 169, 170]. To maximize LNP delivery potential, optimizing their design and precision targeting is essential (Figure 3).

#### 7.1.1 Change p*K*_a_ to Improve Organ Targeting

The apparent acid dissociation constant (p*K*_a_) is a critical physicochemical property for the ionizable head groups in ionizable lipids in LNPs. It indicates the pH at which ionized and unionized groups on the LNPs surface are equal, serving as an indicator of the particle's surface charge and ionic interactions.

The apparent p*K*_a_ influences the degree of ionization and surface charge of LNPs, affecting their stability, potency, and toxicity. Interactions between ionizable lipids and negatively charged plasma proteins and cell membranes largely govern LNP biological behavior [171, 172, 173]. Acid–base titration and the 2‐(p‐toluidino)‐6‐naphthalene sulfonic acid fluorescence assay are common methods to determine the p*K*_a_ of LNPs [174]. Surface charge impacts cellular uptake, endosomal escape, and biodistribution; an optimal p*K*_a_ is vital for effective mRNA therapeutic efficacy. The challenges of cytotoxicity, inefficient endosomal escape, and incomplete mRNA release are closely related to the ionization state of LNPs. Adjusting the apparent p*K*_a_ is crucial for optimizing LNPs for mRNA delivery. Moderna synthesized 30 ionizable lipids, identifying 14 that induced higher neutralizing antibody titers than DLin‐MC3‐DMA, and 4 with superior luciferase expression. This suggests protein expression post‐IM administration is not a definitive predictor of immunogenicity. The ideal p*K*_a_ range for IM administration was determined to be 6.6–6.9. Ultimately, SM‐102 (p*K*_a_ = 6.68) was selected as the optimal ionizable lipid for Moderna's mRNA‐1273 COVID‐19 vaccine [171]. Modifying the p*K*_a_ of LNPs improves targeting to extrahepatic organs, such as the lungs and spleen [175]. LNPs are more efficiently delivered to the liver with a p*K*_a_ of 6.2–6.5, and to the spleen with a p*K*_a_ of 2–6 [176]. LNPs assembled from ionizable lipids (OF‐Deg‐Lin; p*K*_a_ ≈ 5.7) exhibit enhanced splenic targeting [177]. The head group structure of ionizable lipids determines their p*K*_a_. The capacity of LNPs to bind specific serum proteins is determined by their distinct charge, leading to varied biodistribution. Specifically, negative surface charges correlate directly with splenic update [178].

The apparent p*K*_a_ values of polymeric NPs can be adjusted by modifying the chemical monomers, altering the molar ratios of different monomer units within the polymer, or altering the molecular weight of the polymer. For example, a library of ultra‐pH‐sensitive nanoprobes was developed based on the block copolymer PEO‐b‐P(R1‐r‐R2), where R1 and R2 are random monomer units. Polyethylene oxide‐b‐poly(2‐(dibutylamino) ethyl methacrylate) (PEO‐b‐PDBA) and PEO‐b‐PDPA (propyl‐substituted) exhibit apparent p*K*_a_ values of 5.3 and 6.2, respectively. By precisely controlling the molar ratio of DBA to DPA, a series of PEO‐b‐P(DPA‐r‐DBA) copolymers with apparent p*K*_a_ values ranging from 5.3 to 6.2 was synthesized [173, 179]. The apparent p*K*_a_ of LNPs is influenced by the molar ratios of all lipid constituents rather than the p*K*_a_ of a single lipid component. It can be modulated by chemically modifying the head group and hydrophobic tail structures. Thus, the p*K*_a_ of LNPs can be adjusted by chemically modifying the lipid structure or combining two or more lipids with distinct p*K*_a_ values. In this way, blending three structurally similar ionizable lipids—designated as compounds 15 (p*K*_a_ = 5.64), 16 (p*K*_a_ = 6.44), and 17 (p*K*_a_ = 6.93)—generated a set of LNPs with p*K*_a_ values between 5.64 and 6.93. Similarly, a mixture of YSK05 (p*K*_a_ = 6.50) and YSK12‐C4 (p*K*_a_ = 8.00) was used to produce LNPs with p*K*_a_ values adjustable within the range of 6.50–8.00 [180, 181].

The structure and physicochemical properties of LNPs critically impact mRNA delivery. The apparent p*K*_a_ is a reliable parameter for predicting LNP encapsulation efficiency and delivery efficacy. LNPs with p*K*_a_ values between 6 and 7 exhibit the highest potency for RNA delivery. Specifically, p*K*_a_ values of 6.2–6.5 are optimal for siRNA delivery to the liver, while values of 6.6–6.9 enhance immune responses following IM mRNA administration. Thus, finding the optimal p*K*_a_ of an LNP tailored to the target tissue and disease context is crucial, making it difficult to define a universal p*K*_a_ suitable for all RNA delivery applications.

#### 7.1.2 Molecular Modifications to Improve Organ Targeting

In 2020, Siegwart et al. [67, 182] developed a fifth component, selective oran targeting (SORT) molecule to target organs by altering the p*K*_a_ of LNPs, impacting the unique interactions between LNPs and serum proteins. A basic four‐component LNP formulation (5A2‐SC8, DOPE, DMG‐PEG, and cholesterol) was developed to deliver mRNA encoding luciferase protein. High luciferase expression was noted in the livers of BALB/c mice via an imaging system. Incorporating different molar ratios of DOTAP or 1,2‐dioleoyl‐sn‐glycero‐3‐phosphate (18PA) into this formulation led to varied luciferase expression in lungs or spleens of mice. A 50% molar ratio of DOTAP resulted in the highest lung expression, while 30% 18PA enhanced spleen expression. Similar expression patterns were observed after replacing 5A2‐SC8 with DLin‐MC3‐DMA or C12‐200, demonstrating the robustness of the targeting effects. Although total protein content in mice remained, significant differences appeared in the luciferase levels within the lung, spleen, and liver tissues. Furthermore, replacing DOTAP with permanent ionizable lipids like dimethyl dioctadecylammonium (DDAB) or 1,2‐dimyristoyl‐sn‐glycero‐3‐ethylphosphocholine (EPC) retained strong lung‐targeted delivery. Similarly, substituting 18PA with anionic lipids such as 1,2‐dimyristoyl‐sn‐glycero‐3‐phosphate (14PA) or sn‐(3‐oleoyl‐2‐hydroxy)‐glycerol 1‐phospho‐sn‐3′‐(1′,2′‐dioleoyl)‐glycerol (18BMP) enabled efficient spleen‐targeted delivery [166]. This suggests that the physicochemical properties of the SORT component, such as net charge or p*K*_a_, are more crucial for organ tropism than its molecular structure. High‐dose in vivo toxicity assessments of SORT–LNPs showed no changes in renal or hepatic function and no tissue damage following single or repeated administrations. This confirms their safety and low toxicity. Hence, SORT technology represents a major advancement for protein replacement and gene correction therapies, enabling precise treatment of a broad spectrum of diseases. SORT–LNPs can also target the lungs for vaccine delivery, promoting mucosal immune responses and protection against infectious diseases caused by respiratory pathogens.

Siegwart's team [183] has recently advanced on previous SORT LNP research by developing a dual‐targeting LNP platform called dual SORT LNPs. This platform simultaneously delivers gene editors to the liver and lungs. In mouse models, the liver‐targeting SORT–LNPs achieved transfection in up to 90% of hepatocytes, while the lung‐targeting SORT–LNPs transfected approximately 20% of bronchial epithelial cells and 17% of pulmonary monocytes and macrophages. This dual‐delivery method successfully corrected the PiZ mutation in the SERPINA1 gene, responsible for alpha‐1 antitrypsin deficiency (AATD), within hepatic and pulmonary tissues in AATD mouse models, improving organ‐specific disease phenotypes and enhancing neutrophil elastase inhibition in serum and bronchoalveolar lavage fluid. The gene editing effects persisted for up to 32 weeks, demonstrating sustained therapeutic benefits and highlighting the potential for gene therapy targeting multiorgan diseases [183]. However, the chemical properties of SORT significantly enhance the delivery efficiency of LNPs. The novel SORT platform improves selective LNP targeting, advancing mRNA vaccine development for disease prevention and cancer therapy.

The spleen, the largest secondary lymphoid organ in vertebrates, is crucial for immune defense, including mononuclear phagocytosis, blood filtration, and blood storage regulation. Kowalski's research team [184] performed ring‐opening polymerization of lactone and tertiary aminomethyl alcohols to obtain novel ionizable aminopolymer esters (APEs). When blended with LNPs, these APEs exhibit an increased preference for splenic APCs [184]. Currently approved LNP‐based delivery systems, such as Onpattro, BNT162b2, and mRNA‐1273, utilize DLin‐MC3‐DMA, ALC‐0315, and SM‐102, respectively, as their key ionizable lipids. The high delivery efficiency of ionizable lipids has led to the clinical success of these products. However, recent studies show that these commercialized lipids exhibit limited organ‐targeting specificity due to interaction with LDLRs, causing potential hepatotoxicity at high levels [146]. To address this, novel ionizable lipids have been developed to enhance spleen targeting and improve immune responses to prophylactic vaccines, while minimizing off‐target effects in the liver. Meanwhile, Anderson et al. [177] synthesized a series of alkenyl aminolipids (OF‐XX) in vitro via alkenyl epoxide and polyamine core ring‐opening polymerization reactions. Among these, OF‐02 LNPs effectively target the liver. To target the spleen, they synthesized OF‐Deg‐Lin containing a diketopiperazine nucleus and four esterified unsaturated tail chains based on the structure of OF‐02. The resultant LNPs exhibited a targeted preference for splenic B lymphocyte [177]. Another novel lipid (OF‐C4‐Deg‐Lin) synthesized by adding four alkyl groups achieved superior mRNA delivery potential [185]. Gomi et al. [186] created LNPs using acid‐soluble (PS with targeted delivery to the spleen, accelerating mRNA vaccine development. Dong et al. [187] developed a novel LNP (TNT‐b10) derived from the siRNA delivery vector TNT‐a10, with modified functional group location, significantly improving splenic targeting. Wu et al. [188] designed 161 carbonate‐bearing ionizable lipids (CAILs) and formulated them into LNPs capable of efficiently delivering mRNA to splenic natural killer (NK) cells. In the Ai9 mouse model, the optimized CAIL–LNPs achieved successful transfection of 21% of splenic NK cells [188]. Ni et al. [189] prepared piperazine‐containing ionizable lipids with Pi‐A10 to effectively deliver mRNAs to immune cells in the liver and spleen, with 50% of the mRNA expressed in splenic macrophages and 30% in dendritic cells (DCs).

Targeted delivery to DCs is imperative for cancer vaccine research. Veiga et al. [190] effectively activated splenic DCs by reducing ionizable lipids in unmodified RNA–lipid complexes. Meanwhile, Tom et al. [191] found that 30% of the mRNA carried by CD4 antibody‐coupled LNPs was expressed in splenic macrophages. Similar results were reported by Tomácz et al. [192]. In contrast, CD5 antibody‐coupled LNPs preferentially targeted lymph node T‐cells. LNPs modified with an antibody specific for DC205—highly expressed on DCs—are readily detected and taken up by DC205^+^ DC [193]. These studies show that coupling antibodies or ligands to LNPs can specifically target immune cells in immune organs, mimicking the activation of host immune responses to pathogens.

Regarding targeting nonimmune organs, LNPs with amide bonds in the tail of ionizable lipids preferentially target mouse lungs, whereas those with ester bonds in the tail tend to target the liver. Xu et al. [194] determined that by modifying the head structure of LNPs, O‐series LNPs target liver cells, while N‐series LNPs target lung cells. Specifically, 306‐N16B–LNP demonstrate selective mRNA delivery to lung endothelial cells, while 113‐N16B–LNP readily transfect lung endothelial cells, macrophages, and epithelial cells following systemic administration [194]. Additionally, 113‐O12B–LNP deliver tumor antigen mRNA to lymph nodes, inducing a long‐lasting memory response to prevent tumor recurrence [195]. Six novel classes of ionizable lipids (LD, TM, CD, DS, TD, and MK) synthesized from small‐molecule ligands and amino lipids have been formulated to deliver luciferase‐expressing mRNAs with the capacity to cross the blood–brain barrier [196]. Organ targeting by LNPs can also be affected by the proportion of different lipids. Peer et al. [197] increased the proportion of auxiliary lipids to 30%, noting that LNPs, with slightly reduced size and potential biodegradability, and those comprising 30% DSPC and 30% IL‐10, were well tolerated in the colon of a mouse colitis model. This was evidenced by the natural targeting of the colon and poor targeting of the liver and spleen [197]. Additionally, the particle size of LNPs can also influence their targeting capacity [168]. Increasing the size of LNPs to 200 nm by adding salt to LNP buffer improves their delivery to mouse lymphocytes and DCs without altering their specific composition, resulting in immune activation.

Molecular modification methods, incorporating novel lipid components, and coupling antibodies and ligands, as well as altering the particle size, can enhance the organ targeting of LNPs and mitigate hepatotoxic responses. However, quality control of LNPs is crucial due to the potential unknown adverse reactions to newly added components, such as lipids and couplers, and their mechanisms.

#### 7.1.3 Route of Administration

The administration route of mRNA–LNP vaccines impacts their biodistribution and cellular uptake, as well as the type, intensity, and duration of the immune response. The most common injection routes include IM, IV, intradermal (ID), subcutaneous (SC), and intranodal (IN) administration. Alternative delivery routes include intranasal, intravaginal, and intratumoral injections. Given that immune cells and lymphoid organs are the primary targets of vaccination, the anatomical and physiological characteristics of the administration site—such as skin, muscle, lymphoid tissue, and systemic circulation—profoundly affect vaccine safety and efficacy. ID injection delivers mRNA vaccines directly into the dermal layer, which is composed of connective tissue rich in APCs, such as DCs and macrophages. These cells internalize and process the mRNA vaccine. Additionally, the dense vascular and lymphatic networks within the dermis facilitate the transport of the vaccine and APCs to the draining lymph nodes, activating T and B cells and eliciting a robust immune response [198, 199, 200, 201, 202]. However, ID injections are also associated with a higher risk of local adverse reactions, including swelling, pain, erythema, and pruritus. SC injection delivers the vaccine to the SC layer, primarily comprising loose adipose tissue and fewer immune cells than the dermis. SC administration allows a larger injection volume and typically results in milder pain, although the vaccine absorption tends to be slower (Figure 3).

The muscle tissue targeted by IM injection lies deeper than the dermis and SC layers and is characterized by an extensive vascular network that helps recruit and recirculate immune cells, including APCs [203, 204, 205]. Radioactively labeled mRNA has been detected at the injection site and in draining lymph nodes for up to 28 h post‐IM administration, generally causing milder local side effects than ID and SC injections [206]. IN injections deliver mRNA vaccines directly into peripheral lymphoid organs, allowing immediate interaction with APCs and resident T and B cells. This method is highly effective due to the direct targeting of lymphoid tissue but often requires ultrasound guidance, making it complex and less common in clinical trials [207]. IV administration delivers mRNA vaccines directly into systemic circulation. LNPs administered via this route are predominantly taken up by the liver, including hepatocytes, Kupffer cells, and liver sinusoidal endothelial cells. IV injection may also facilitate the direct delivery of mRNA vaccines to circulating immune cells and lymphoid organs, thereby significantly enhancing immunogenicity. However, plasma proteins, enzymes, and shear stress in the bloodstream can interfere with delivery efficiency. Moreover, systemic exposure may lead to unintended adverse effects, such as splenic damage and lymphocyte depletion [208, 209].

Mucosal lymphoid tissues also contain abundant APCs; mucosal administration can be achieved via intranasal or intravaginal routes. Intranasal administration targets the nasal mucosa and nasal‐associated lymphoid tissue and may allow mRNA to reach the lungs via the trachea. Immature and activated APCs in the lungs can process the mRNA vaccine [210, 211]. Meanwhile, intravaginal delivery can transport mRNA vaccines to infection sites and induce the expression of neutralizing antibodies. For instance, intravaginal administration of mRNA encoding anti‐HIV antibodies elicits robust antibody expression in the reproductive tracts of sheep and rhesus macaques. The expressed antigens were predominantly distributed in cervical epithelial and stromal cells [212]. In addition to directly modifying LNPs, local administration can enhance the targeting ability of LNPs. In preclinical and clinical trials, LNPs are commonly administered via SC, ID, or IM injections to target nearby draining lymph nodes and activate immune responses [79, 213, 214]. However, most mRNA vaccines are administered intramuscularly. For tumor vaccines, nasal drops and intratumoral administration can improve efficacy [215, 216]. For instance, mRNA‐2416 expresses OX40L following intratumoral administration in patients with solid tumors (NCT03323398). Meanwhile, mRNA‐2752 encodes OX40L, IL‐23, and IL‐36γ, to activate the proinflammatory tumor microenvironment by enhancing T‐cell proliferation and immune memory (NCT03739931). In a related study, Ghosh et al. [217] reported the effective delivery of nebulized LNPs (F2, F8, F11, and F17) to the lungs of mice. This highlights the potential of personalized medicine, including the administration route, to optimize drug delivery to target organs according to the specific disease and desired therapeutic outcomes.

In summary, local administration of mRNA vaccines restricts their biodistribution to and accumulation in vital organs, such as the liver, heart, and brain, mitigating the risk of systemic toxicity, including hepatotoxicity and inflammation. After injection, APCs at the site take up the vaccine and migrate to regional lymph nodes to activate T and B cells and enhance immunogenicity. This method also improves LNP delivery efficiency and mRNA translation. In contrast, systemic delivery (e.g., IV injection) can cause widespread distribution, nonspecific antigen expression, and unintended immune activation, making local administration preferable for current mRNA vaccines.

### 7.2 Endosomal Escape

LNPs primarily enter cells via endocytosis, requiring mRNA molecules to escape from nuclear endosomes into the cytoplasm to be translated into proteins. However, most mRNAs are transported by endosomes to lysosomes for degradation, with only a small fraction escaping to the cytoplasm. This represents a significant impediment to drug delivery development [218, 219, 220, 221]. Enhancing drug therapeutic efficacy necessitates a profound understanding of LNP interactions with in vivo pathways. This will aid in designing optimal drug therapeutics, allowing more mRNA molecules to escape endosomes. Two theories currently explain the endosomal escape process.

- As early nuclear endosomes evolve into lysosomes, the pH level changes from 7.4 in the normal physiological environment to 6.5 in early nuclear endosomes, 6 in late nuclear endosomes, and 5 in lysosomes [222]. Endosomal escape by LNPs is primarily associated with the pH‐dependent membrane phase. Ionizable lipids are pivotal in dictating the fundamental properties of LNPs and closely correlate with the mechanism of endosomal escape [223]. When the hydrophobic end of the ionizable lipid is equivalent in size to the head group, LNPs often disperse in saline as a bilayer [112]. Once internalized by the target cells, nuclear endosomes engulf the LNPs. As the nuclear endosome matures and its pH decreases, the pH‐sensitive ionizable lipid head group carries a positive charge, inducing H_II_ structure formation after interacting with the negatively charged nuclear endosome membranes. This causes membrane damage, releasing the loaded drug into the cytoplasm.
- Another prevalent mechanism of endosomal escape is the “proton sponge effect” [224]. In the endosomal environment, LNPs carry a positive charge, causing chloride ions to passively diffuse into the nuclear endosomal compartment to achieve membrane equilibrium. This increases osmotic pressure within the endosome, leading to swelling and subsequent rupture, facilitating nucleic acid escape.

Current studies primarily focus on the efficiency of endosomal escape in relation to transfection capacity, while relatively less attention is given to the migration of labeled nucleic acid molecules from endosomes to the cytoplasmic lysate. Although modifying the structure of LNPs can improve endosomal escape, no comprehensive solution has been defined. Thus, exploring viable methods is necessary to achieve optimal LNP endosomal escape (Figure 3).

The size of the hydrophobic group in ionizable lipids influences endosome escape. Unsaturated bonds in the hydrophobic tail create a spatial barrier via the rigid structure of the cis‐double bond, facilitating the formation of the H_II_ phase and aiding endosome escape [225]. Sato et al. [226] developed a pH‐responsive ionizable lipid library, with CL1H6 demonstrating high endosomal escape efficiency due to its hydrophilic head group with a tertiary amine and two tails with unsaturated bonds. The head group affects the p*K*_a_ of LNPs, enhancing endosomal escape [226]. Tanaka et al. [227] developed a self‐degradable lipid molecule that accelerates LNP decomposition, promoting mRNA release into the cytoplasm. Hence, incorporating a hydrophobic tail with unsaturated bonds can improve endosomal escape, potentially increasing with additional unsaturated bond [227]. Based on the four‐component LNP, Syn–LNP was assembled from the ionizable lipid L319 and intramuscularly administered to C57BL/6 mice, significantly improving endosomal escape and mRNA delivery [228]. Moreover, terminally branched ionizable lipids (BEND lipids) were synthesized. Following transfection of HeLa cells, the longest and shortest terminally branched BEND lipids exerted the greatest effects [228]. Furthermore, a mouse model revealed that ionizable lipids E4t‐494 and E6t‐494 exhibit superior delivery performance [229]. Hence, incorporating branched chains at the terminal end of ionizable lipids can effectively enhance LNP's membrane‐disrupting capacity and endosomal escape.

Although PEGylated materials prolong the blood circulation time of LNPs, they also create a protective barrier due to the shielding effect of PEG, hindering LNP binding to target cells and reducing their uptake. Specifically, PEG compromises the ability of LNPs to fuse with cellular and nuclear endosomal membranes, impeding the release of loaded drugs into the cytoplasm of target cells. To address these limitations, we developed pH‐sensitive PEGylated lipids that undergo cleavage under reducing conditions, improving LNP endosomal escape and enhancing therapeutic efficacy and safety [230, 231]. The LNP surface was modified with anti‐HER2 F(ab′) antibody fragments or PEI polymers, along with lipids, peptides, and other endosome‐interacting molecules. The optimized LNPs exhibited significantly improved endosome escape [232, 233, 234].

Cholesterol's structural properties also impact the endosomal escape ability of LNPs. Introducing eLNP composed of C‐24 alkyl phytosterols into LNPs significantly improves their gene transfection ability. Unlike conventional spherical LNPs, the polyhedral structure of eLNPs enhances cellular uptake and endosomal escape [235]. Substituting cholesterol with naturally occurring phytol enhances LNPs delivery. Similarly, substituting with hydroxycholesterol influences cholesterol recognition by NPC1, improving delivery efficiency [236, 237]. Furthermore, small molecule manipulation of the endosomal recycling pathway can enhance transfection efficiency. For example, annexin A6 (ANXA6) regulates the endocytic pathway, membrane reorganization, and vesicle transport through its lipid‐binding activity on Ca^2+^. However, inhibiting ANXA6–lipid interactions may promote mRNA release from endosomes. Indeed, NAV and ES5—small molecules that specifically inhibit ANXA6–lipid interactions—significantly enhance LNP the delivery efficiency by 1.5‐2‐fold [238]. Furthermore, NPC1 is pivotal in regulating the primary pathway of siRNA delivery. In NPC1‐deficient cells, target genes are silenced, facilitating LNP endosomal escape [239]. Additionally, NPC1 binds to cholesterol in the endocytic pathway, impeding endosomal escape [240].

The structural characteristics of LNP are pivotal in determining its functional capabilities. The incorporation of unsaturated tail groups into ionizable lipids, the introduction of environmentally responsive PEGylation, the addition of polymers and antibodies to enhance membrane rupture, and the improvement of LNP's ability to escape endosomes through active modulation of endosomal recycling pathways provide a theoretical foundation for subsequent studies.

### 7.3 Controlling Adverse Reactions

The success of LNP‐based mRNA vaccines against COVID‐19 is a major milestone in infectious disease prevention. As clinical applications expand to cancer, rare genetic disorders, autoimmune conditions, and cardiovascular diseases, safety concerns have become increasingly prominent. Key safety issues include the intrinsic immunogenicity of LNPs, hepatotoxicity, and potential inflammatory responses.

#### 7.3.1 Immunogenicity

LNPs generally exhibit favorable safety profiles in clinical trials, particularly in the context of mRNA vaccines such as BNT162b2 and mRNA‐1273. The most common adverse events are mild to moderate, including injection pain, fatigue, and fever, primarily attributed to the activation of innate immune pathways by the ionizable lipids within LNPs. Approximately 15% of younger participants in mRNA‐1273 trials reported high‐grade fevers (>39°C), generally resolving within 48 h [3, 4]. Activation of the innate TLR4/NF‐κB signaling pathway promotes proinflammatory cytokine secretion, including IL‐6 and IL‐1β. This pathway exhibits adjuvant‐like properties, enhancing adaptive immune responses by stimulating CD4⁺ T cell activation and promoting the Th1 immune profile, contributing to the overall vaccine efficacy [241].

While rare, notable safety concerns have emerged, such as myocarditis and anaphylaxis, particularly in younger males and those with preexisting allergies. The BNT162b2 vaccine has been associated with a higher risk of myocarditis, with a risk ratio of 3.24 (95% CI, 1.55–12.44) and an absolute risk difference of 2.7 cases per 100,000 individuals (95% CI, 1.0–4.6). This risk is substantially elevated following SARS‐CoV‐2 infection [242]. A case report highlighted a healthy 24‐year‐old male who developed myocarditis 5 days after receiving a second dose of the mRNA‐1273 vaccine, with elevated troponin (18.94 ng/mL; normal: 0.01–0.04), C‐reactive protein (26.4 mg/L; normal: <10.0), and creatine kinase (704 U/L; normal: 49–348). Given negative SARS‐CoV‐2 PCR and serological testing, the myocarditis was suspected to be associated with the LNP component of the vaccine [243], as anti‐PEG antibodies induced hypersensitivity reactions in approximately 2.3 cases per million doses administered. Despite these events, mRNA‐1273 and BNT162b2 vaccines remain effective and immunogenic. The CDC's Advisory Committee on Immunization Practices concluded that the benefits of COVID‐19 vaccination outweigh the potential risks [244]. By December 23, 2020, 1,893,360 doses of Pfizer's mRNA vaccine were administered in the United States to protect against SARS‐CoV‐2 infection, with 4,393 adverse events reported the Vaccine Adverse Event Reporting System. Of these, Although the underlying causes are not fully elucidated, but 175 were potential severe anaphylactic reactions possibly linked to the LNP component [245, 246]. Ndeupen et al. [247] demonstrated that the LNPs in preclinical nucleoside‐modified mRNA vaccines induce severe inflammation in mice, with massive neutrophil infiltration and inflammatory cytokine/chemokine secretion. Immunization of rabbits and mice with PEG‐coupled proteins and Fuchsin's adjuvant induced secretion of anti‐PEG antibodies and anti‐PEG IgM through the TLR7 signaling pathway. Additionally, administration of LNPs with TLR agonists induced high levels of anti‐PEG antibodies and allergic reaction [248, 249, 250]. PEG‐induced complement‐activated hypersensitivity and IgE antibodies were detected in LNP‐injected mice, with inflammatory reactions developing after repeated administration. The interaction between anti‐PEG antibodies and LNPs reduces the delivery efficiency through the accelerated blood clearance (ABC) phenomenon driven by activation of Fcγ receptors on intrinsic immune cells by anti‐PEG antibodies [251, 252, 253]. Notably, anti‐PEG antibodies have been observed to be associated with anaphylactic reactions, which can result in severe allergic response [252, 253, 254]. The severity of allergic reactions is directly proportional to the percentage of PEG in the LNP formulation [255]. Consequently, judicious selection of polymers to replace or modify PEGs may mitigate the adverse reactions associated with LNP administration.

Dong et al. [256] substituted PEGylated lipids with polyketide lipids, substantially enhancing LNP delivery efficiency, with a comparable safety profile in C57BL/6J mice. Piel et al. [257] synthesized poly(N‐methyl‐N‐vinylacetamide) lipids, which upon secondary administration to zebrafish and mouse models, prolonged LNP circulation time, reduced immune responses, and did not induce a systemic proinflammatory response. More recently, Zhao et al. [258] demonstrated that branched cleavable PEG derivatives, such as branched PEG 240k, can lower levels of anti‐PEG IgM antibodies without triggering the ABC phenomenon. Similarly, replacing PEG with poly(sarcosine) (Psar) significantly decreases anti‐Psar IgM and IgG levels in Wistar rats [259]. A recent study by Jiang's research team [260] at Cornell University indicated that synthesizing polycarboxybetaine (PCB) lipids with varying molecular weights using reversible addition–fragmentation chain‐transfer (RAFT) polymerization can enhance the performance of LNPs. Under identical lipid formulations containing MC3, SM‐102, and ALC‐0315, the M2‐PCB lipid (*m* ≈ 5, *n* = 12) notably improves endosomal escape efficiency and exhibits robust target gene expression in vitro in THP‐1, HeLa, and RAW264.7 cell lines. Replacing PEG with PCB significantly enhanced the efficacy of mRNA‐based therapeutics and markedly reduced their immunogenicity. In vivo immunological studies confirmed that PCB‐containing LNPs achieve greater therapeutic outcomes and induce a Th1‐biased immune response in C57BL/6 mouse models. Moreover, PCB demonstrates favorable antioxidative safety in hemolysis assays. PCB–LNPs induce fewer antipolymer IgM antibodies than PEG‐containing LNPs, effectively mitigating the ABC effect associated with PEGylation. Thus, PCB–LNPs can be repeatedly administered intravenously without compromising therapeutic efficacy [260]. Feng et al. [261] utilized Evans blue‐modified lipids to replace PEGylated lipids in the construction of LNPs. This modification markedly reduced hepatic accumulation of mRNA vaccines, while conferring strong antitumor and antiviral efficacy. The engineered LNPs also elicited robust cytotoxic T lymphocyte activation and neutralizing antibody production, underscoring their high therapeutic efficiency and favorable safety profile [261]. Hence, selecting appropriate alternative polymers to PEG can reduce LNP‐associated adverse reactions. Structurally modifying PEG can also modulate immune‐related adverse reactions. For example, selecting hydroxy PEG over methoxy PEG reduces PEG antibody secretion [262]. Moreover, PEG introduces amino or carboxyl groups that facilitate targeted ligand coupling and reduce self‐induced immunogenicit [263]. Branch‐modified PEG2k–DAG significantly attenuates the ABC phenomenon in animal models by reducing the antibody‐binding site through a spatial site‐blocking effect [264]. The dynamic shedding of PEG through environmentally responsive bonds (e.g., disulfide bonds and matrix metallase‐sensitive linkages) reduces long‐term PEG exposure and decreases anti‐PEG antibody production [265, 266]. Additionally, immunizing BALB/c mice with gangliosides coupled with PEG significantly reduces anti‐PEG IgM production [267]. Therefore, the proportion of PEGylated lipids in LNPs should be strictly controlled to reduce adverse reactions.

#### 7.3.2 Hepatotoxicity

Following adsorption of ApoE, LNPs are taken up by hepatocytes via LDLRs, leading to hepatic accumulation. Ionizable lipids may contribute to hepatotoxicity by accumulating in lysosomes and affecting mitochondrial membrane potential. Acute drug‐induced liver injury is typically assessed by measuring plasma levels of alanine aminotransferase (ALT), aspartate aminotransferase (AST), and alkaline phosphatase [268].

In preclinical studies, high‐dose IV administration of mRNA–LNP formulations induced mild lymphocyte depletion adjacent to the central splenic artery in approximately 80% of mice in the LNP control group, indicative of a formulation‐related effect. Meanwhile, in a study evaluating the efficacy of an LNP‐based therapy for ASA, hepatic accumulation of submicron‐sized lipid droplets was observed in the LNP control group [269]. In rabbits administered a single IM injection of an mRNA–LNP vaccine encoding the influenza hemagglutinin H3 antigen, increased serum AST, ALT, and C‐reactive protein levels were observed [270]. Histopathological analysis revealed hepatic vacuolar degeneration, infiltration of inflammatory cells and erythrocytes, and heightened germinal center activity within the spleen. Similarly, murine models subjected to high doses (>3 mg/kg) of the ionizable lipid DLin‐MC3‐DMA demonstrated hepatic vacuolization and necrosis [271].

The use of biodegradable lipids such as L319 significantly mitigate these toxic effects. The chemical structure of ionizable lipids directly influences LNP safety and targeting specificity. Incorporating pH‐sensitive linkages, such as esters or ketals, into the hydrophobic tails of ionizable lipids effectively reduces toxicity [173]. L319 contains ester bonds and degrades into low‐toxicity alcohols and acids in the acidic lysosomal environment, with substantially lower hepatotoxicity than nondegradable DLin‐MC3‐DMA. Other innovations include β‐thioester bonds cleaved by lysosomal thioesterases and disulfide‐bridged lipids metabolized intracellularly.

Extrahepatic targeting has emerged as a core strategy for reducing liver‐associated toxicity [272]. For example, spleen‐specific targeting can enhance the immunogenicity of prophylactic vaccines by promoting protective immune responses. Moreover, cell‐specific delivery that activates CD8⁺ T cell responses may improve therapeutic efficacy. Cen et al. [271] at the Institute of Medicinal Biotechnology, Chinese Academy of Medical Sciences, developed a ketal‐linked ionizable lipid (KEL) containing biodegradable ketal and ester moieties. Through iterative optimization of the head and tail groups to adjust p*K*_a_ and molecular geometry, (4S)‐KEL12 was identified as a safe and effective ionizable lipid. Mortality was not observed in high‐dose (3 mg/kg) single‐injection studies in male rats, with low serum ALT/AST levels. KEL12 also exhibited excellent biodegradability; no lipid accumulation was detected in major organs (heart, liver, lungs, kidneys) within 120 h of post‐IM injection. Compared with SM‐102, KEL12 demonstrated markedly reduced hepatic accumulation [271]. Additionally, strategies such as reducing PEG–lipid content (<1 mol%) and incorporating anionic phospholipids (e.g., DOPG) increase APC uptake of LNPs [273].

#### 7.3.3 Inflammation

The safety profile of LNPs in COVID‐19 vaccines may differ when injected via other administration routes or incorporated in nonvaccine applications. Regardless of the administration route, LNPs can activate the innate immune system, leading to the release of proinflammatory cytokines and promoting the infiltration of activated leukocytes into injured tissues [274]. While essential for adaptive immunity development, excessive innate immune stimulation can induce immunotoxicity and dysregulate immune cell behavior. Although LNPs share certain mechanistic features with traditional adjuvants regarding immunostimulatory activity, their inherent immunogenicity presents a major challenge for expanding the application of mRNA–LNP formulations beyond prophylactic vaccines, particularly in the context of repeated dosing and long‐term safety. Moreover, these proinflammatory effects may pose significant risks for patients with underlying inflammatory conditions. As such, careful balance must be struck during LNP design to retain beneficial adjuvant activity while minimizing adverse reactions associated with ionizable lipid‐induced inflammation.

Researchers at the University of Pennsylvania examined lipid structures and signaling pathways underlying LNP‐induced inflammation. Ionizable lipids, such as C12‐200, 98N12‐5, and cKK‐E12, were found to elicit robust cytokine responses, including IL‐1α, IL‐6, TNF‐α, IFN‐β, and MCP‐1 due to endosomal membrane damage during mRNA escape. Large‐scale endosomal rupture recruits galectins (e.g., Galectin‐8), which mediate inflammation cascades and facilitate autolysosomal degradation of damaged endosomes. Meanwhile, smaller‐scale membrane disruptions are primarily resolved through the ESCRT (endosomal sorting complexes required for transport) machinery. Interestingly, the ionizable lipid 4A3‐SC8 efficiently delivers mRNA without inducing high levels of proinflammatory cytokines. Immunostaining for ALG‐2‐interacting protein X, a component of the ESCRT pathway, indicated that 4A3‐SC8 promotes minor endosomal escape without triggering inflammation. Furthermore, thiodigalactoside—a small‐molecule galectin inhibitor—suppressed LNP‐induced inflammatory responses [275]. This study established a positive correlation between the degree of endosomal escape and the severity of adverse inflammatory reactions.

Patel et al. [276] further demonstrated that plasmid DNA (pDNA)‐loaded LNPs activate the cyclic GMP‐AMP synthase–stimulator of interferon genes (cGAS–STING) pathway, leading to pronounced inflammatory responses. Preclinical models showed LNP–pDNA markedly increased serum IL‐6 and TNF‐α levels within 6 h of IV administration, leading to a type I interferon storm. However, coadministration of nitro‐oleic acid (NOA—an endogenous STING inhibitor lipid—significantly attenuated these systemic inflammatory responses [276]. Prostaglandin E2 analogs also suppress inflammasome activation. Macrophage‐derived exosome‐enveloped LNPs may also mitigate adverse effects by exploiting CD47 on exosomes to evade mononuclear phagocyte system clearance [277]. Meanwhile, surface adhesion molecules, such as lymphocyte function‐associated antigen 1 (LFA‐1), enhance targeting to inflamed tissues. Collectively, these findings underscore the need for refined LNP designs to balance delivery and immunotoxicity [278]. Additionally, residual organic solvents (e.g., chloroform and ethanol) or impurities created during synthesis can disrupt cell membranes, triggering local inflammation, inducing ROS production, and causing DNA or protein damage. Large particles and high surface charges can also impact biodistribution, triggering hepatotoxicity or splenomegaly. Furthermore, specific populations, such as HLA‐B*15:02 allele carriers, exhibit heightened sensitivity to carbamazepine lipids, are predisposed to severe cutaneous reactions, including Stevens‐Johnson syndrome and toxic epidermal necrolysis [279].

In current clinical trials of LNP‐based therapeutics, the most frequently reported adverse effects are predominantly attributed to the intrinsic immunogenicity, hepatotoxicity, and proinflammatory responses associated with LNPs. The mechanisms underlying LNP‐induced immunogenicity and inflammation share signaling pathways, highlighting the importance of future LNP design strategies to preserve adjuvant‐like immunostimulatory effects while minimizing excessive inflammatory responses. For instance, substituting or optimizing PEGylated lipids may provide a means to fine‐tune these effects [267]. LNP accumulation in the liver is also common and can compromise the pharmacodynamic potential of the delivered payload and, in some cases, lead to hepatotoxicity. Therefore, the development of next‐generation ionizable lipids must place greater emphasis on tissue‐specific targeting to therapeutic sites while minimizing off‐target accumulation in metabolically active organs, such as the liver, to reduce the risk of metabolic disorders [270].

Efficient endosomal escape is critical for the functional delivery of mRNA therapeutics; however, the resultant endosomal membrane damage serves as a source of inflammation, with the degree of damage varying by formulation. Future research should focus on designing multitailed and biodegradable lipids (e.g., 4A3‐SC8) that support controlled endosomal escape and recruit endogenous repair mechanisms, such as the ESCRT complex, to limit inflammation [260]. As this platform is refined, LNPs have the potential to contribute to global health by providing safe, effective, and versatile nucleic acid delivery for various therapeutic domains such as cancer immunotherapy (e.g., CAR‐T therapy, CRISPR‐mediated gene editing), rare diseases (e.g., protein replacement therapy), autoimmune disorders, and cardiovascular conditions. Following the structural optimization of LNPs, it is essential to establish an effective quality control system to ensure clinical consistency and safety.

## 8 Quality Control of LNPs

Presently, there is a lack of consensus regarding the quality standards for LNPs, and testing methods vary among research institutes and enterprises. The expanding application of LNPs across various fields has diversified quality standards, complicating research, development, production, and quality control. Table 3 summarizes recommended testing methods and CQAs from various pharmacopoeias.

**TABLE 3:** LNP quality control items and their analytical detection methods.

Quality	Attribute	Suggested analytical approaches	References
Physical and chemical properties	Appearance Subvisible particles	ChP<0904>, USP <1> EP <2.9.2>, JP< 6.06>	/
	LNP particle size	DLS, TEM, SEM, AFM, NTA SAXS, SANS	[280, 281, 282, 283]
	Polydispersity (PDI)	DLS	[284]
	Zeta potential	ELS, DLS	[285]
Indicators of effectiveness	Encapsulation rate	RiboGreen/Triton‐100	[286]
	mRNA integrity	Sanger sequence, HTS, RT‐PCR	[287]
	mRNA content	UV, qPCR, ddPCR	[288]
	Empty particle ratio	CICS	[289]
	Lipid composition analysis	RP‐HPLC‐CAD, ChP<0512>, EP <2.2.46>	/
Safety	Endotoxin	ChP<1143>, USP <85>, EP <2.6.14>, JP<2.2>	/
	Sterility	ChP<1101>, USP <71> EP <2.6.1>, JP< 4.06>	/
	Abnormal toxicity	ChP<1141>	/
Other	pH	ChP<0631>, USP <791> EP <2.2.3>, JP<2.54>	/
	Residual solvent	ChP<0861>, USP<467>, EP< 5.4>, JP<2.46>	/
	Osmolality	ChP<0632>, USP<785>	/
	Insoluble particles	ChP<0903>, USP<787>, EP<2.9.19>, JP<6.07>	/
	Lyophilized dosage forms‐moisture	ChP<0832>, USP<921>, EP<2.2.13>	/

Abbreviations: AFM: atomic force microscope, ChP: Pharmacopoeia of the People's Republic of China, CICS: cylindrical illumination confocal microscopy, ddPCR: droplet digital polymerase chain reaction, DLS: dynamic light scattering, ELS: electrophoretic light scattering, EP: European Pharmacopoeia, HTS: high‐throughput screening, JP: The Japanese Pharmacopoeia, NTA: nanoparticle tracking analysis, PCR, RT‐PCR: reverse transcription‐polymerase chain reaction, PDI: polymer dispersity index, qPCR: quantitative real‐time RP‐HPLC‐CAD: reversed‐phase high‐performance liquid chromatography with charged aerosol detector‐charged aerosol detector, SANS: small‐angle neutron scattering, SAXS: small angle X‐ray scattering, SEM: scanning electron microscope, TEM: transmission electron microscope, UV: ultraviolet, USP: United States Pharmacopoeia.

For potential impurities arising during the production process, it is essential to substantiate the critical value to ensure suitability for testing and alignment within the clinical acceptance range. In the case of potential exogenous factors, supporting evidence and risk assessment results must be provided to demonstrate their removal or control [290]. The Center for Drug Evaluation of the National Drug Administration, in its “Technical Guidelines for Pharmaceutical Research and Evaluation of In Vitro Gene Modification Systems (Trial),” stipulates that nonclinical lipid studies must consider the inherent toxicity of lipids and the potential for antibody induction. Additionally, in August 2024, the CDE issued the “Technical Guidelines for Nonclinical Studies of Prophylactic mRNA Vaccines” (Draft for Public Comment) and “The Technical Guidelines for Research” (Draft for Comment), which mandate genotoxicity testing if an mRNA vaccine contains novel lipids or excipients. Moreover, in the context of the initial utilization of novel raw materials, the “Guidelines for Non‐clinical Safety Evaluation of New Pharmaceutical Excipients” (CDE) explicitly stipulates that the mechanism of action, synthetic purification process, quality control, supplier information, production batch, and safety of new lipid materials must be thoroughly researched and documented.

Quality studies on LNPs must align with the CQAs of traditional nanomedicines, as CQAs are essential for ensuring consistency in product quality across batches. Cryo‐electron microscopic observation of mRNA‐loaded LNPs identified two predominant morphologies: a single‐compartment configuration with a smooth surface lacking vesicle protrusion and a two‐compartment or multicompartment composite structure with double‐layer vesicle protrusions. The morphology of mRNA–LNPs is influenced by various factors during preparation, including lipid composition, flow rate ratio, and type and concentration of buffer solutions. Throughout the process, mRNA–LNP samples are stored and utilized; repeated freeze–thaw cycles, freeze–drying, and re‐solubilization can alter their morphology.

Most LNP products in clinical studies and the market are single‐compartmental structures. Cryo‐electron tomography showed that LNPs in sodium citrate buffer at pH 4 form vesicles with a Bled structure. Multicompartmental vesicles can improve in vitro transfection and in vivo protein expression in mice [291]. LNP particle size is heterogeneous and unstable during production and storage due to variations in reparation processes and equipment. For instance, in microfluidics, higher total flow rates and increased ratios of aqueous to lipid phases decrease LNP particle size.

For microfluidic chips used in LNP encapsulation, GMP/US FDA‐approved sanitary metal materials, such as 316 L, are recommended. When plastic materials are required for the chip, they must comply with CDE's “Guidelines for Systematic Compatibility Studies of Plastic Components Used in the Manufacture of Injectable Chemical Drugs (for Trial Implementation).” These guidelines stipulate that the suitability of a plastic chip component system must be evaluated through risk assessments and comprehensive compatibility studies. Sahay et al. [292] determined that LNP particle size increases markedly after lyophilization in HEPES and PBS buffers but remained unchanged in Tris buffer. The CDE advises determining the mean particle size of LNPs, cumulative distribution (*D10, D50, D90*), and total particle count to achieve stable particle sizes. Encapsulation rate is a key attribute of LNP quality, indicating the proportion of effective drug loading.

Subtle variations in the lipid composition of LNPs can influence their physicochemical properties. The ratio of ionizable lipids to mRNA (e.g., nitrogen‐to‐phosphorus ratio) should be monitored. Moreover, the various preparation processes applied for LNPs can impact quality consistency. Ensuring consistent quality during the scale‐up process is also challenging due to variations in equipment and process parameters. To ensure consistent quality, it is imperative to optimize the equipment type, improve control strategies for the process parameters, establish a comprehensive process analysis system, and employ additional methodologies to ensure quality consistency. Manufacturing instruments must also comply with the “Guide to Good Manufacturing Practice” for effective processes and uniform products.

Per the World Health Organization's “Guidelines on transmissible spongiform encephalopathies in relation to biological and pharmaceutical products,” the source and purity of selected lipids must be characterized. As the delivery system for mRNA vaccines, characterizing the composition of LNPs is critical for understanding its potential systemic toxicity and genotoxicity. Furthermore, the excipients that may affect the safety and efficacy of the vaccine during development must be considered. Pardi et al. [293] formulated LNPs encoding influenza virus and SARS‐CoV‐2 target proteins. The LNPs promote follicular helper T‐cell and memory B‐cell responses with superior adjuvant enhancement to MF59‐like AddaVax. The adjuvant activity of LNPs may be dependent on ionizable lipid components and IL‐6 induction [293]. Bett et al. [294] demonstrated that LNPs significantly enhance the total B‐cell immune response to influenza virus and SARS‐CoV‐2 antigens tested and that ionizable lipids and their cholesterol in the LNP lipid mixture act as vaccine adjuvants, activating the intrinsic immune response.

Physical, chemical, and structural characterization of LNPs is necessary for preclinical assessment of the quality, efficacy, and safety of drugs. The utility of analytical methods for LNP characterization can facilitate rapid development of innovative RNA therapeutics. For example, after encapsulating a loaded drug, purification via TFF is imperative to remove unencapsulated drugs and free lipid materials. In this process, ultrafiltration membranes with an appropriate molecular weight cutoff are typically selected to ensure the encapsulated complexes do not pass through.

A recent publication by the United States Pharmacopoeia (USP), titled “Analytical Procedures for the Quality of mRNA Vaccines” (2024), offers a compelling illustration of this process. Meanwhile, “Quality of mRNA Vaccines and Therapeutics‐Draft Guidelines,” provides a comprehensive overview of the procedures and guidelines for ensuring the quality of mRNA vaccines and therapeutics [295]. However, limitations in current detection techniques, such as dynamic light scattering, persist, including errors in measuring the particle size distribution in polydisperse systems, which are not ideal for quality control during large‐scale production. Moreover, cryo‐transmission electron microscopy can only qualitatively detect the local field of view, impacting subjectivity. Given the plethora of reports pertaining to adverse reactions to mRNA vaccines, there is a compelling rationale for the enhancement of CQA detection methodologies for LNP, thereby providing a robust theoretical framework to support regulatory authorities.

Despite advances in quality control that enhance the reproducibility of LNP formulations, unresolved issues persist when progressing toward clinical use. Concerns related to safety, scalable manufacturing, and regulatory evaluation remain critical obstacles, underscoring the need to address these clinical translation barriers before realizing the potential of LNPs.

## 9 Clinical Translation Barriers

### 9.1 Safety Considerations

Safety remains one of the most pressing challenges for the successful clinical translation of LNP‐based therapeutics. While the global rollout of mRNA–LNP vaccines has demonstrated that these systems can be safe and well tolerated in the short term, their broader use in chronic diseases and repeated dosing scenarios exposes a range of potential toxicological concerns [296]. One of the most widely discussed issues is the acute activation of the innate immune system, which may occur through pattern recognition receptors such as TLRs or the complement system. This can manifest as systemic inflammatory responses or, in severe cases, complement activation‐related pseudoallergy [204]. In addition, hepatotoxicity has been observed in some preclinical studies, raising concerns about the accumulation and metabolism of ionizable lipids in the liver [297]. Nonbiodegradable lipid components pose further risks, as repeated administration may lead to their persistence in tissues, resulting in long‐term off‐target effects [298]. Another barrier is the immunogenicity associated with PEGylated lipids. Although PEG is often employed to enhance circulation time, repeated dosing can elicit anti‐PEG antibodies, which accelerate blood clearance and may induce hypersensitivity reactions, thereby compromising both safety and efficacy [299].

These challenges are particularly relevant for indications requiring chronic treatment, such as genetic diseases or cancer immunotherapy, where frequent administration is unavoidable [300]. Moreover, data on long‐term safety remain scarce, especially in vulnerable populations such as children, the elderly, and immunocompromised patients [301]. Addressing these issues requires the rational design of biodegradable lipids that can be safely metabolized, coupled with systematic toxicological assessments in relevant animal models. Development of predictive biomarkers for adverse events and improvements in preclinical models—such as organ‐on‐chip platforms or humanized models—may provide deeper insights into the mechanisms of toxicity and immunogenicity. Overall, the establishment of a robust safety profile will be essential to broaden the therapeutic potential of LNP‐based medicines beyond vaccines.

### 9.2 Manufacturing Challenges

Efficient, scalable, and reproducible manufacturing represents another critical barrier for the translation of LNP‐based therapeutics. The formulation of LNPs typically relies on rapid mixing methods, such as microfluidics or ethanol injection, which enable precise control of NP size and encapsulation efficiency at laboratory scale [302]. However, scaling up these processes to industrial production remains a significant challenge. Consistency in particle size distribution, encapsulation efficiency, and surface properties must be maintained across large batches to meet regulatory standards, but variability in raw materials—particularly in ionizable lipids, and nucleic acid payloads—can compromise reproducibility [303]. Moreover, manufacturing of GMP‐grade ionizable lipids is costly and technically demanding, with complex purification steps that increase production costs and limit global accessibility. Another practical obstacle lies in the supply chain and logistics. Most currently approved mRNA–LNP vaccines require stringent cold‐chain storage and transport conditions, with temperatures as low as −70°C, posing serious challenges for distribution, especially in low‐ and middle‐income countries [304]. Although advances in lyophilization, thermostable LNP formulations, and alternative storage buffers are emerging, their application in large‐scale commercial products remains limited [305].

To overcome these hurdles, a shift toward robust quality‐by‐design strategies is needed, integrating advanced process analytical technologies (PAT) to ensure real‐time monitoring and control of CQAs. Additionally, automation, high‐throughput formulation platforms, and closed‐system bioprocessing are being explored to reduce variability and improve scalability. Collaborative efforts between academia, industry, and regulatory bodies are also necessary to establish standardized manufacturing protocols and benchmarks that can accelerate technology transfer from the laboratory to GMP facilities. Ultimately, addressing these manufacturing barriers is essential not only for cost‐effectiveness and commercial viability but also for ensuring equitable global access to LNP‐based therapeutics.

### 9.3 Regulatory Hurdles

The regulatory pathway for LNP‐based therapeutics remains complex and relatively underdeveloped compared with traditional drug modalities, posing a significant barrier to clinical translation. Unlike small molecules or conventional biologics, LNPs are hybrid systems that combine a therapeutic payload (such as mRNA, siRNA, or protein) with a nanostructured delivery vehicle, meaning that both the cargo and the carrier must be evaluated simultaneously. Regulatory agencies such as the US FDA and the European Medicines Agency (EMA) currently require detailed physicochemical characterization of LNP formulations, including size, polydispersity, charge, encapsulation efficiency, and stability. In addition, comprehensive evaluation of biodistribution, clearance pathways, and potential immunogenicity are mandated. However, standardized assays and validated analytical methods for NP evaluation remain limited, creating variability between studies and complicating regulatory submissions [306]. Another challenge lies in the regulatory evaluation of novel lipid components. Many ionizable lipids and helper lipids designed for next‐generation LNPs are chemically unique and lack historical safety data, making it difficult to classify them as conventional excipients. This uncertainty can lead to delays in approval, as additional toxicological and pharmacokinetic studies are often required. Furthermore, guidance for combination products—where the therapeutic activity results from the interplay between the drug payload and its carriers still evolving, leaving sponsors with limited clarity on submission strategies.

Beyond technical hurdles, global harmonization of regulatory frameworks is lacking. Differences in requirements between the US FDA, EMA, and regulatory bodies in Asia create additional barriers for multinational clinical development. To address these issues, international collaboration will be crucial in establishing harmonized guidelines and reference standards. Regulatory innovation, such as adaptive approval pathways and rolling review processes, may also accelerate clinical translation while ensuring patient safety. In the long term, fostering stronger dialogue between regulators, researchers, and industry stakeholders will be essential for balancing innovation with rigorous safety and efficacy evaluation.

In summary, despite remarkable progress in advancing LNP‐based therapeutics, unresolved challenges in safety, manufacturing, and regulatory frameworks continue to impede their broad clinical application. Addressing these translational barriers through innovative design, standardized production, and harmonized oversight will be pivotal for unlocking the full potential of LNPs. Looking ahead, future research directions offer promising strategies to overcome these limitations.

## 10 Conclusion and Future Outlook

LNPs, as delivery vehicles for mRNA vaccines and therapeutic drug, have achieved significant breakthroughs, particularly in the prevention of SARS‐CoV‐2 infection. Through structural design and component screening, LNPs have been shown to enhance nucleic acids and proteins stability, biocompatibility, tunability, and suitability for large‐scale manufacturing. Initially, ionizable lipids, comprising specific saturated alkyl chain tail formulations (ALC‐0315 and SM‐102), auxiliary phospholipids, cholesterol, and PEGylated lipids were synthesized [307]. However, the successful launch of BNT162b2 and mRNA‐1273 vaccines provided substantial evidence for the feasibility and effectiveness of LNPs in mRNA vaccines and therapeutic. However, certain challenges must be overcome. LNP‐based drugs primarily rely on passive targeting, leading to drug accumulation in the liver and potential hepatotoxicity, which hinders systemic efficacy [187]. After injection, the LNP‐encapsulated molecules enter cells via endocytosis but become largely degraded in lysosomes, with only a small proportion escaping to be translated into proteins in the cytoplasm [218, 219]. Allergic reactions are also a concern, as PEG in LNPs can trigger anti‐PEG antibody secretion, compromising delivery efficiency due to the ABC phenomenon, and activating complement through the classical pathway [248, 250, 252]. Finally, the uneven morphology and endosomal escape leads to cell damage and triggers varying degrees of inflammatory responses, and repeated freeze–thaw cycles of LNPs can increase particle size and affect mRNA vaccine quality [291, 292].

To address these challenges, researchers have improved the targeting ability of LNPs by altering the p*K*_a_ value, adopting degradable linkage bonds, optimizing the ionizable lipid structures, incorporating different types of alkyl tails, modifying or substituting LNP components with polymers, coupling antibodies or ligands to the surface of LNPs, and varying administration routes. Enhancing endosomal escape involves promoting H_II_ phase formation and using environment‐responsive PEG. Immunogenicity and toxicity can be reduced by optimizing lipid composition, developing new ionizable lipids, and modifying or replacing PEG lipids. Further investigation into the long‐term safety and metabolic pathways of LNPs is also warranted to improve biocompatibility with endogenous lipid analogs or biomimetic membranes [31]. For resource‐poor areas, LNP formulations should include protective agents or high‐temperature resistant lipids for room temperature storage. Although there are effective experimental solutions to the current challenges, with the advent of the information age, the optimization strategy of LNP can be combined with big data models.

Given the rapid advancement of LNP technology, future research should further explore its applications in precision drug delivery. Recent advancements in artificial intelligence (AI)‐driven LNP design employ graph neural networks, gradient‐boosting algorithms, and transformer‐based models to predict key physicochemical properties (e.g., apparent p*K*_a_, transfection efficiency) and enable high‐throughput virtual screening. Wang et al. [308] trained models to forecast both p*K*_a_ and delivery efficacy, yielding novel ionizable lipids superior to DLin‐MC3‐DMA validation. Transformer‐based frameworks such as TransLNP outperformed conventional graph neural nets in large‐scale lipid design tasks [309]. Additionally, Cai and Shubhra [310] demonstrated translational utility of AI‐designed LNPs specifically for pulmonary mRNA delivery. Platforms such as GENESIS288—a multimodal embodied AI system—enable predictive modeling of lipid cargo interactions, membrane fusion, and NP biodistribution, thereby complementing traditional all‐atom and coarse‐grained simulations [311]. LNP technology is progressing toward subcellular‐level delivery precision. scRNA‐seq and spatial transcriptomics provide detailed insights into the heterogeneity of cell types and receptor expression profiles within target tissues, assisting in optimizing surface modifications on LNPs. Recent scRNA‐seq analyses of the tumor microenvironment have identified distinct receptor expression patterns—such as CD206 on tumor‐associated macrophages (TAMs) and αvβ3 integrin on DCs—enabling precise targeting via ligand‐functionalized LNPs. For instance, mannose‐modified LNPs achieve selective delivery to CD206⁺ TAMs and DCs in vivo, while RGD‐conjugated LNPs preferentially bind αvβ3‐rich DCs [312, 313, 314]. Organ‐on‐chip technologies provide a biomimetic platform for in vitro screening and mechanistic studies of LNPs, particularly in recapitulating complex organ microenvironments such as the blood–brain barrier, hepatic sinusoids, and pulmonary alveoli. For example, in a humanized liver‐chip model, differential uptake of various LNP formulations by hepatocytes and Kupffer cells can be systematically evaluated. These studies support developing LNPs capable of nonimmune organ‐specific delivery, expanding the application of mRNA technology to hepatic metabolic diseases and rare genetic disorders.

In personalized cancer immunotherapy, whole‐exome sequencing or RNA‐seq of patient tumor samples can identify unique somatic mutations. Bioinformatics tools such as NetMHCpan predict neo‐antigenic epitopes arising from these mutations, while LNPs deliver mRNA encoding the neoantigens, which are subsequently presented via the MHC class I pathway to activate cytotoxic CD8⁺ T cells for targeted tumor cell killing. The central role of LNPs in delivering mRNA vaccines is shifting the paradigm of cancer therapy from nonspecific cytotoxic approaches to precise immune activation. Overall, the LNP platform is being refined within an AI‐driven “design–screen–validate” loop, merging nanomaterials, immunology, and cellular biology. Engineered LNPs offer multifunctional, intelligent, and reliable platforms that hold great promise for advancing precision medicine, particularly in targeted drug delivery and disease‐specific interventions. This integration is transforming LNPs from vaccine carriers to broad‐spectrum therapeutic tools. In particular, the application of LNPs in cancer immunotherapy, autoimmune diseases, and gene editing (e.g., CRISPR/Cas9 mRNA delivery) underscores their emergence as a cornerstone of precision medicine. Emerging strategies, including ligand modification, stimuli‐responsive release, and integration with AI‐driven modeling platforms, offer promising avenues for enhancing LNP performance and accelerating their translation into precision medicine.

## Author Contributions

Conceptualization: Xiaochi Li, Aihua Zhao, and Miao Xu. Data collection: Xiaochi Li, Junli Li, Jiazheng Wei, Weixin Du, Cheng Su, and Xiaobing Shen. Writing—original draft preparation: Xiaochi Li. Writing—review and editing: Xiaochi Li, Junli Li, Aihua Zhao, and Miao Xu. All authors have read and agreed to the published version of the manuscript.

## Ethics Statement

The authors have nothing to report.

## Conflicts of Interest

The authors declare no conflicts of interest.

## Data Availability

The authors have nothing to report.
